# Olanzapine induces Mdivi-1-resistant disruption of insulin signaling in skeletal muscle cells

**DOI:** 10.3389/fphar.2026.1920978

**Published:** 2026-10-26

**Authors:** Pablo Arias, Catalina Salamanca, Fernanda Briones-Manriquez, Lucas Oyaneder, Andrea Marimán, Gonzalo Almarza, Edson Yañez, Juan Rodriguez, Rodrigo Troncoso, Leonel E. Rojo, Carlos F. Lagos, Roberto Bravo-Sagua, Andrea del Campo

**Affiliations:** 1 Laboratorio de Fisiología y Bioenergética Celular (LABFBE), Facultad de Química y de Farmacia, Pontificia Universidad Católica de Chile, Santiago, Chile; 2 Laboratorio de Investigación en Nutrición y Actividad Física (LABINAF), Instituto de Nutrición y Tecnología de Los Alimentos (INTA), Universidad de Chile, Santiago, Chile; 3 Departamento de Biología, Facultad de Química y Biología, Universidad de Santiago de Chile (USACH), Santiago, Chile; 4 Centro de Biotecnología Acuícola, Universidad de Santiago de Chile (USACH), Santiago, Chile; 5 Chemical Biology & Drug Discovery Lab, Escuela de Química y Farmacia, Facultad de Ciencias, Universidad San Sebastián, Santiago, Chile; 6 Centro Basal Ciencia & Vida, Fundación Ciencia & Vida, Santiago, Chile; 7 Laboratorio de Obesidad y Metabolismo (OMEGA), Instituto de Nutrición y Tecnología de Los Alimentos (INTA), Universidad de Chile, Santiago, Chile; 8 Advanced Center for Chronic Diseases (ACCDiS), Universidad de Chile & Pontificia Universidad Católica de Chile, Santiago, Chile; 9 Centro Interuniversitario de Envejecimiento Saludable (CIES), Consorcio de Universidades del Estado de Chile (CUECH), Santiago, Chile; 10 International Research Center for Medical Sciences, Kumamoto University, Kumamoto, Japan

**Keywords:** insulin resistance, Mdivi-1, mitochondria, olanzapine, skeletal muscle

## Abstract

**Introduction:**

Second‐generation antipsychotics such as olanzapine (OLZ) are widely prescribed because they produce fewer extrapyramidal adverse effects than first‐generation agents. However, OLZ is strongly associated with metabolic dysfunction, and alterations in mitochondrial dynamics have been proposed to contribute to these effects. We investigated whether OLZ‐induced mitochondrial dysfunction is linked to impaired insulin signaling in skeletal muscle cells and explored a potential interaction with the mitochondrial fission regulator dynamin‐related protein 1 (DRP1).

**Methods:**

L6 skeletal myoblasts were treated with OLZ, alone or in combination with the mitochondrial fission inhibitor Mdivi‐1. Insulin signaling, glucose uptake, mitochondrial morphology, and insulin‐stimulated mitochondrial bioenergetics were evaluated. In parallel, molecular dynamics simulations and MM/PBSA calculations were performed using a common DRP1/Mg^2^^+^ receptor model to compare the interactions of protonated OLZ and Mdivi‐1 within the DRP1 GTPase domain.

**Results:**

OLZ reduced insulin‐mediated signaling and glucose uptake, promoted mitochondrial fragmentation, and impaired the insulin‐induced increase in mitochondrial bioenergetic activity. Co-treatment with Mdivi‐1 partially rescued OLZ‐induced mitochondrial fragmentation but did not restore insulin responsiveness. Molecular simulations showed that protonated OLZ and Mdivi‐1 remained within the same hydrophobic region of the DRP1 GTPase domain and shared an anchoring interaction involving Ile252. Their calculated MM/PBSA interaction free energies were similar (–22.26 ± 1.7 and –24.50 ± 0.5 kcal mol^−^^1^, respectively), supporting compatible occupancy of a shared binding region but not demonstrating high‐affinity or GTP‐competitive binding.

**Discussion:**

These findings indicate that OLZ disrupts mitochondrial dynamics and bioenergetics while decreasing insulin sensitivity in skeletal muscle cells. However, the dissociation between recovery of mitochondrial morphology and persistence of insulin resistance suggests that excessive mitochondrial fission alone does not account for OLZ‐induced metabolic dysfunction. The computational results are compatible with an interaction of OLZ with the DRP1 GTPase domain but do not establish DRP1 as its primary pharmacological target. OLZ‐associated insulin resistance therefore likely involves additional, potentially extramitochondrial mechanisms.

## 1 Introduction

The rising consumption of second-generation antipsychotics (SGAs) such as olanzapine (OLZ) has become a major contributor to metabolic syndrome, a cluster of comorbidities that increases the risk of cardiovascular disease, stroke, and type 2 diabetes (Neeland et al., 2024). SGAs are widely known to induce insulin resistance (IR) and disrupt glucose homeostasis independently of caloric intake (Bernardo et al., 2021; Rognoni et al., 2021; Sneller et al., 2021; Stogios et al., 2022; Mortimer et al., 2023; Liao et al., 2026). However, the metabolic liability of SGAs is not uniform. Clozapine and OLZ consistently exhibit the highest risk of weight gain, hyperglycemia, and insulin resistance, whereas agents such as aripiprazole, ziprasidone, amisulpride, and lurasidone represent a substantially lower metabolic burden (Leucht et al., 2013; Burschinski et al., 2023). This marked gradient suggests that SGA-induced metabolic dysfunction is driven in a compound-specific manner rather than via a class-wide mechanism, highlighting the importance of elucidating the molecular basis underlying the particularly high metabolic liability of OLZ (Fitzgerald et al., 2021; Wang et al., 2022).

Interestingly, the two SGAs with the highest metabolic liability, clozapine and OLZ, are structurally related dibenzodiazepine derivatives that share a broadly overlapping receptor-binding profile (Junior et al., 2015). Consistent with this pharmacological similarity, clozapine has been reported to induce mitochondrial depolarization, ROS accumulation, and mitochondrial fragmentation in several cellular models (Contreras-Shannon et al., 2013; Shao, 2022), suggesting that mitochondrial dysfunction may represent a common feature of these structurally related antipsychotics. Skeletal muscle is a primary target of this metabolic toxicity due to its central role in insulin-stimulated glucose uptake and oxidation (Hulett et al., 2022). Mechanistically, OLZ directly impairs insulin signaling in L6 muscle cells, leading to defective glycogen synthesis and decreased glucose transport (Engl et al., 2005). This highlights skeletal muscle dysfunction as a key early event linking SGA use to systemic metabolic decline.

Disruption of the insulin signaling cascade and mitochondrial dysfunction are tightly intertwined in the development of abnormal metabolic responses in skeletal myocytes. In early stages of IR, patients show a reduction in the oxidative phosphorylation activity of mitochondria (Gilbert, 2021). Moreover, in advanced stages of metabolic syndrome, obese and diabetic patients display a decreased activity of the tricarboxylic acid cycle and respiratory chain, along with a combination of mitochondrial DNA damage and reduced mitochondrial DNA content (Sangwung et al., 2020). Regarding mitochondrial morphology, patients with obesity and/or type 2 diabetes (T2D) present a decrease in the mitochondrial volume of skeletal muscle cells, accompanied by a loss in functionality (Rocha et al., 2020). Additionally, it has been described that the abrogation of mitochondrial fusion by silencing either Mitofusin two or Optic atrophy 1 (OPA1) produces a decrease in insulin-induced Akt phosphorylation and glucose uptake in L6 skeletal muscle cells (del Campo et al., 2014). Furthermore, recent evidence suggests that mitochondrial network fragmentation may be involved in the pathogenesis of T2D and IR (Vezza et al., 2022; García-Peña et al., 2024). However, despite the clear relationship between metabolic syndrome and mitochondrial dysfunction, there is little evidence showing alterations on the mitochondrial network of patients being treated with SGAs.

Previously, we reported a potential interaction between OLZ and the Insulin receptor, which may be associated with its detrimental metabolic effect through dampening of the downstream signaling (del Campo et al., 2018); however, this may not be its only mechanism of action. Indirect evidence suggests that the metabolic effects of OLZ may be mediated by fragmentation of the mitochondrial network (del Campo et al., 2018). Similarly, recent studies demonstrated that clozapine, another SGA, alters mitochondrial morphology and reduces ATP levels in insulin-responsive cells in a dose-dependent manner *in vitro* (Bagherlou, 2025; Fiorani et al., 2025). On the other hand, a recent study described that inhibition of mitochondrial fragmentation with the pharmacological Dynamin-related protein 1 (Drp1) inhibitor Mdivi-1 restores insulin signaling in cellular models of T2D (Lin et al., 2018). Also, inhibition of mitochondrial fission reportedly improves muscle insulin signaling and systemic insulin sensitivity in obese mice (Kugler et al., 2023; Lin et al., 2024).

Thus, our study aims to evaluate whether preventing mitochondrial fragmentation with Mdivi-1 in the skeletal myocyte cell line L6 prevents OLZ-induced mitochondrial fragmentation and insulin resistance, thus restoring glucose homeostasis.

## 2 Materials and methods

### 2.1 Cell culture

L6 muscle cells were obtained from ATCC (ATCC CRL-1458) and cultured as a myoblast monolayer as described (del Campo et al., 2014). Prior to all experiments, cells were cultured in α-MEM (#12000-022, Gibco, USA) supplemented with 10% FBS (FBS-12A, Capricorn Scientific, USA) for 2 days, then maintained in α-MEM 2% FBS for the next 2 days.

For experimentation, cells were left untreated (control condition) or treated with either 160 μM OLZ (Olanzapine Zyprexa® injectable solution 10 mg/mL, Lilly, USA), or 10 μM Mdivi-1 (M0199, Sigma, USA), or both for 24 h and then, under serum-free conditions were left untreated or exposed to 100 nM insulin (C143945C (Humalong), Lilly, USA) for 30 min. The 160 μM OLZ concentration was selected based on previously published work where OLZ-induced metabolic dysfunction was assessed in immortalized cell lines (del Campo et al., 2021). On the other hand, the 10 μM Mdivi-1 concentration was selected because it is widely used to inhibit Drp1-mediated mitochondrial fission while remaining non-cytotoxic in cultured cells (Cassidy-Stone et al., 2008; Kim et al., 2013).

### 2.2 Cell viability assay

Cells were cultured at 60% confluence in 24-well plates then treated as aforementioned. After experimentation, cells were loaded with 1 μg/mL propidium iodide (P3566, ThermoFisher, USA) for 5 min at room temperature. Afterwards, cells were detached with trypsin (TRY-1B10, Capricorn Scientific, USA), and fluorescence was assessed by flow cytometry using a 543 nm/560 nm excitation/emission filter (Accuri C6, BD Biosciences, Franklin Lakes, NJ, USA). A minimum of 10,000 events were acquired per condition per experiment. Each condition was assessed in duplicate (technical replicates).

### 2.3 Western blot analysis

Cells were cultured at 80% confluence in 6-well plates then treated as aforementioned. After experimentation, cells were homogenized in lysis buffer containing 20 mM Tris-HCl solution (pH 7.5) containing 1% Triton X-100, 1 mM EDTA, 1 mM EGTA, 20 mM NaF, 1 mM Na_2_P_2_O_7_, 10% glycerol, 140 mM NaCl, 10 mM Na_3_VO_4_, 1 mM PMSF and protease inhibitor cocktail (11,836,170,001, Sigma-Aldrich, St. Louis, MO, USA). Subsequently, lysates were centrifuged at 15,000 rcf for 10 min at 4 °C to eliminate debris. Protein concentration was determined by the bicinchoninic acid (BCA) assay (23225, Thermo Fisher Scientific, Walthan, MA, USA). 25 μg of protein were separated by sodium dodecyl sulfate-polyacrylamide gel electrophoresis (SDS-PAGE) and electrotransferred onto PVDF membranes. Samples were then blocked with TBS containing 0.05% Tween-20 (TBS-T) and 5% milk. Protein detection was carried out by sequential incubation with primary antibodies (1:1,000) overnight at 4 °C followed by secondary antibody staining (1:10,000) for 1 h at room temperature. The primary antibodies used were directed against pSer^473^-Akt (#9271, Cell Signaling, USA), total Akt (2920S, Cell Signaling, USA), Thr^202^/Tyr^204^-ERK1/2 (#4370S, Cell Signaling, USA), total ERK1/2 (#9102S, Cell Signaling, USA) and Vinculin (#13891S, Cell Signaling, USA). The secondary antibodies used were directed against either rabbit IgG (ab6721, Abcam, USA) or mouse IgG (ab6728, Abcam, USA) according to the primary antibody isotype. Chemiluminescent signals were detected using the ECL Supernova kit (XLS3, Cyanagen, Bologna, Italy) and quantified via scanning densitometry (C-DiGit Blot Scanner, LICOR, USA).

### 2.4 Glucose uptake assay

Cells were cultured at 60% confluence in 24-well plates then treated as aforementioned. After experimentation, cells were loaded with 400 nM 2NBDG (N13195, ThermoFisher, USA) in Krebs solution (pH 7.4) consisting in 140 mM NaCl, 5 mM KCl, 3.5 mM CaCl_2_, 2 mM MgCl_2_ and 10 mM HEPES without glucose for 30 min at 37 °C. Afterwards, cells were detached with trypsin (TRY-1B10, Capricorn Scientific, USA), and fluorescence was assessed by flow cytometry using a 488 nm/530 nm excitation/emission filter (Accuri C6, BD Biosciences, Franklin Lakes, NJ, USA). A minimum of 10,000 events were acquired per condition per experiment. Each condition was assessed in duplicate (technical replicates).

### 2.5 Oxygen consumption

Cells were cultured at 80% confluence in 100-mm plates and treated. After experimentation, cells were detached with trypsin then centrifuged at 600 rcf and resuspended in PBS. The cell suspension was then placed in a Clark’s electrode chamber (Yellow Springs Instruments, Yellow Springs, OH, USA) and oxygen levels were measured polarographically at 25 °C and normalized by protein content.

### 2.6 Mitochondrial transmembrane potential evaluation

Cells were cultured at 60% confluence in 24-well plates and treated as aforementioned. After experimentation, cells were loaded with 200 nM TMR (T668, Invitrogen, USA) for 30 min at 37 °C. Afterwards, cells were detached with trypsin, and fluorescence was assessed by flow cytometry using a 543 nm/560 nm excitation/emission filter (Accuri C6, BD Biosciences, Franklin Lakes, NJ, USA). A minimum of 10,000 events were acquired per condition per experiment. Each condition was assessed in duplicate (technical replicates). As a positive control for decreased mitochondrial potential, cells were treated with 50 µM CCCP for 30 min.

### 2.7 ATP quantification

Cells were cultured at 80% confluence in 96-well plates and treated as aforementioned. After experimentation, intracellular ATP content was determined using the Cell Titer Glow assay (G7570, Promega, Madison, WI, USA) following the manufacturer’s instructions. In brief, cells were washed twice with PBS and left with 20 µL of PBS. Immediately after, an equal volume of CellTiter-Glo Reagent was added to each well to induce cell lysis and initiate the luciferase-catalyzed luminescent reaction. Then, the mix was transferred to opaque plates and incubated in the dark at room temperature for 10 min to stabilize the luminescent signal. Signals were measured in a microplate reader BioTek Cytation 5 (Agilent Technologies, Santa Clara, CA, USA). Each condition was assessed in triplicate (technical replicates).

### 2.8 Mitochondrial morphology determination

Cells were cultured at 30% confluence in 24-well plates and treated as aforementioned. After experimentation, cells loaded with 400 nM Mitotracker Green FM (M7514, Thermo Fisher Scientific) for 20 min at 37 °C protected from light. Subsequently, they were washed with Krebs solution (pH 7.4) consisting in 140 mM NaCl, 5 mM KCl, 3.5 mM CaCl_2_, 2 mM MgCl_2_, 10 mM HEPES and 5.6 mM glucose. Cells were maintained in Krebs solution for imaging. Fluorescent microscopy images were captured at 60x with the GFP filter (469 nm/525 nm) of the widefield fluorescence microscope Cytation5 (Agilent Technologies, Santa Clara, CA, USA). Images were acquired as single focal planes using identical exposure time, LED intensity, and camera settings for all experimental groups. Bright-field images of the cells are displayed in Figure 1.

**FIGURE 1:**
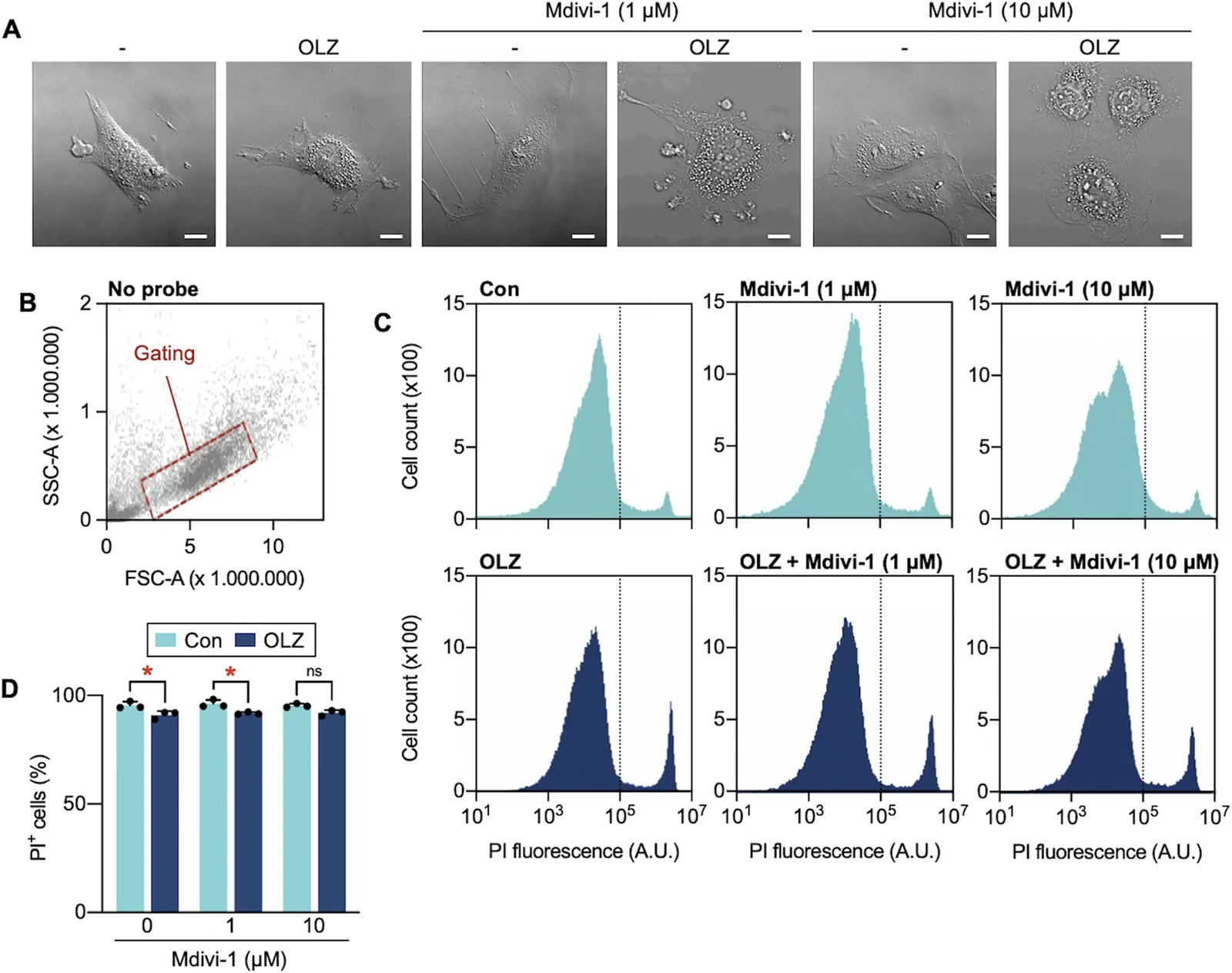
OLZ reduces cell viability in the L6 muscle cell line, which is rescued by Mdivi-1 treatment. L6 myoblasts were incubated with 160 µM OLZ and/or with different concentrations of Mdivi-1 (1 or 10 µm) for 24 h, then photographed or subjected to a propidium iodide (PI) incorporation assay via flow cytometry. **(A)** Representative bright-field microscopy photographs of the cultured cells. **(B)** Representative side-forward scatter dot plot used for initial gating in flow cytometry. **(C)** Representative histograms of the PI incorporation assay in the different conditions. **(D)** Quantification of cell viability of the PI incorporation assay (n = 3). Con: control, OLZ: olanzapine. Results are shown as mean ± SEM. Since the data passed the Shapiro-Wilk normality test, they were analyzed via two-way ANOVA followed by a Bonferroni post-test. *p < 0.05 and ns: not significant. Bar: 10 µm.

Images were analyzed according to the method presented by Valente et al. (2017), using the ImageJ software (NIH, USA). In brief, individual cells were cropped from the images to analyze their whole mitochondrial networks individually, then sharpness, contrast and background noise were adjusted, then images were converted into binary for analysis using the MiNA plug-in, which reports the mitochondrial morphology in terms of individuals and branched networks. Around 30 cells per condition were analyzed from a minimum of 10 randomly selected microscopy fields per independent sample.

### 2.9 In silico molecular docking, molecular dynamics simulations, and MM/PBSA binding free energy calculations

The crystal structure of the human Drp1 (dynamin-1-like) GTPase domain in complex with the non-hydrolysable GTP analogue GMP-PNP (PDB: 4H1V; 2.3 Å resolution) was used as the receptor (Wenger et al., 2013a). A structural model of the GTPase domain (residues 1–320) bearing GTP and Mg^2+^ was prepared in UCSF ChimeraX: hydrogen atoms were added, ligand charges were assigned with the AM1-BCC scheme, and the protein was treated with the AMBER14FB force field. Receptor preparation and docking were performed using OpenEye Toolkit (OpenEye, Cadence Molecular Sciences Inc.), and the binding site was defined as a box of ∼8,000 Å^3^ (20 × 16 × 22 Å) centered on the center of mass of GTP using the OEDOCKING interface. Additionally, a blind docking was performed using FRED v4.3.5.0 over the entire surface of the Drp1 GTPase domain to evaluate the preferred binding site of olanzapine. The resulting binding poses were ranked according to the ChemGauss4 scoring function, and the top-ranked solutions were analyzed for their spatial distribution across the protein surface. Four ligands were evaluated—Mdivi-1, Drpitor1a, DRP1i27 and olanzapine (the last in both neutral and protonated forms). Ligand structures were drawn in Marvin Sketch (ChemAxon Inc.), converted to three-dimensional conformers (1,000 per ligand) with OMEGA v7.0.0.0, and AM1-BCC charges assigned using QUACPAC v2.3.0.0. Conformer ensembles were docked with FRED v4.3.5.0 (Fast Rigid Exhaustive Docking, OpenEye) using the ChemGauss4 scoring function (McGann et al., 2003), generating 100 solutions per ligand that were refined by local energy minimization and clustered to select representative poses (Hawkins et al., 2010; McGann, 2012), and ligand binding affinity predictions were performed using the PRODIGY-LIG web server (Vangone et al., 2019).

To evaluate the dynamic stability and thermodynamics of small-molecule binding within the DRP1 GTPase domain, docked complexes of olanzapine (evaluated in both its unprotonated neutral state, q = 0, and its physiologically predominant piperazine-protonated cationic state, q = +1), Mdivi-1 (q = 0), and reference inhibitors Drpitor1a (q = 0) and DRP1i27 (q = +1) were merged onto a standardized crystallographic human DRP1 receptor base (Chain A, residues 1–321) co-crystallized with catalytic Mg^2+^ (PDB ID: 4H1V), alongside native GTP (q = −4) as a positive control. Small molecules were parameterized with GAFF2 and AM1-BCC partial atomic charges enforcing exact integer net charges, DRP1 was assigned AMBER ff14SB parameters (Maier et al., 2015), GTP polyphosphate was modeled with Meagher parameters (Meagher et al., 2003), and Mg^2+^ was treated with Li/Merz 12-6 parameters (Li et al., 2013). Each complex was solvated in a truncated octahedral TIP3P water box (minimum 1.2 nm buffer) with 0.15 M NaCl using the Joung–Cheatham parameters (Joung and Cheatham, 2008), energy-minimized using steepest descent (Fmax <500 kJ/mol/nm on unconstrained solute heavy atoms), and equilibrated under NVT (100 ps, 310.15 K) and NPT (100 ps, 1.0 bar, Monte Carlo barostat) ensembles with harmonic position restraints (1,000 kJ/mol/nm^2^) on solute heavy atoms. Unrestrained production molecular dynamics (MD) simulations were executed for 10.0 ns per complex (60.0 ns aggregate cohort) using the OpenMM (Eastman et al., 2024) CUDA platform on dual NVIDIA Tesla T4 GPUs with Hydrogen Mass Repartitioning (HMR, m_H = 3.024 amu, 4.0-fs timestep), PME electrostatics (1.0-nm cutoff), and coordinate archiving every 10 ps (1,000 production frames per system). Trajectories were periodic-boundary-unwrapped and Cα-superposed onto the crystallographic reference. End-state MM/PBSA continuum solvation calculations were conducted using AmberTools MMPBSA.py across 200 evenly spaced snapshots (50-ps stride across all 10.0 ns) at physiological ionic strength (0.15 M NaCl) evaluating both solute interior dielectrics ε_in_ = 2.0 and ε_in_ = 4.0 (ε_ext_ = 80.0), with per-residue binding free energy decomposition (idecomp = 1). Statistical standard errors were calibrated using integrated autocorrelation times (N_eff_ = N/2τ_int_) via automated Sokal windowing and cross-validated with Flyvbjerg–Petersen blocking (Flyvbjerg and Petersen, 1989). Pairwise contrasts between inhibitors were evaluated using two-tailed Welch’s t-tests on effective sample sizes with Benjamini–Hochberg False Discovery Rate (FDR) multiplicity adjustments. Protein–ligand interactions were profiled with PLIP (Protein–Ligand Interaction Profiler) (Salentin et al., 2015). Figures were rendered using PyMOL v3.1.8 (Schrödinger, LLC).

### 2.10 Statistical analysis

Data are presented by means ± standard error (bars) of the individual values per group (points). Data were tested for normality using the Shapiro-Wilk test. Most of the groups passed the normality test and their means were compared using two-way ANOVA followed by a Bonferroni’s post-test. This post-test was preferred because of its simplicity and wide use. For the groups that did not pass the normality test, i.e., phospho-Akt analysis (Figure 2B) and ATP levels (Figure 3E), their means were compared using the Kruskal-Wallis test followed by a Dunn’s post-test. This post-test was preferred because of its simplicity and wide use. Statistical analyses were conducted using GraphPad Prism version 9 (GraphPad Software Inc., San Diego, USA). A p-value <0.05 was considered statistically significant, corresponding to a 95% confidence interval.

**FIGURE 2:**
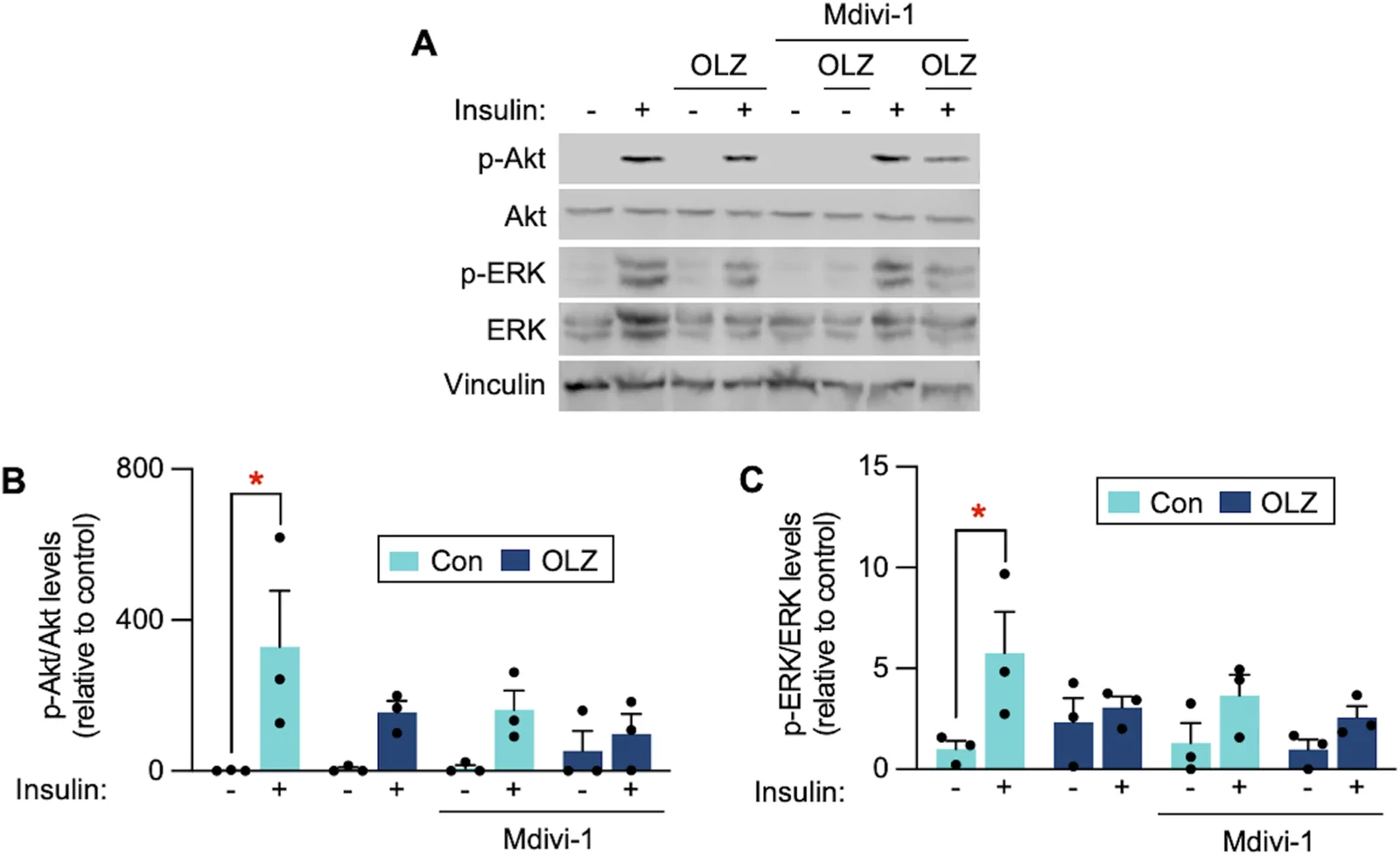
Both OLZ and Mdivi-1 disrupt insulin signaling in the L6 muscle cell line. L6 myoblasts were incubated with 160 µM OLZ and/or 10 µM Mdivi-1 for 24 h, then subjected to a 30 min treatment with 100 nM insulin. **(A)** Representative densitometries of insulin-stimulated Akt Ser^473^ phosphorylation in L6 cells, assessed through Western blot. **(B)** Quantification of p-Ser^473^ Akt phosphorylation normalized by total Akt evaluated as in panel A (n = 3). **(C)** Quantification of p-Thr^202^/p-Tyr^204^ ERK phosphorylation normalized by total ERK evaluated as in panel C (n = 3). Con: control, OLZ: olanzapine. Results are shown as mean ± SEM. Since the data of the phospho-Akt analysis did not pass the Shapiro-Wilk normality test, groups’ means were compared using the Kruskal-Wallis test followed by a Dunn’s test. Data of the phospho-ERK passed the normality test and were analyzed via two-way ANOVA followed by a Bonferroni post-test. *p < 0.05.

**FIGURE 3:**
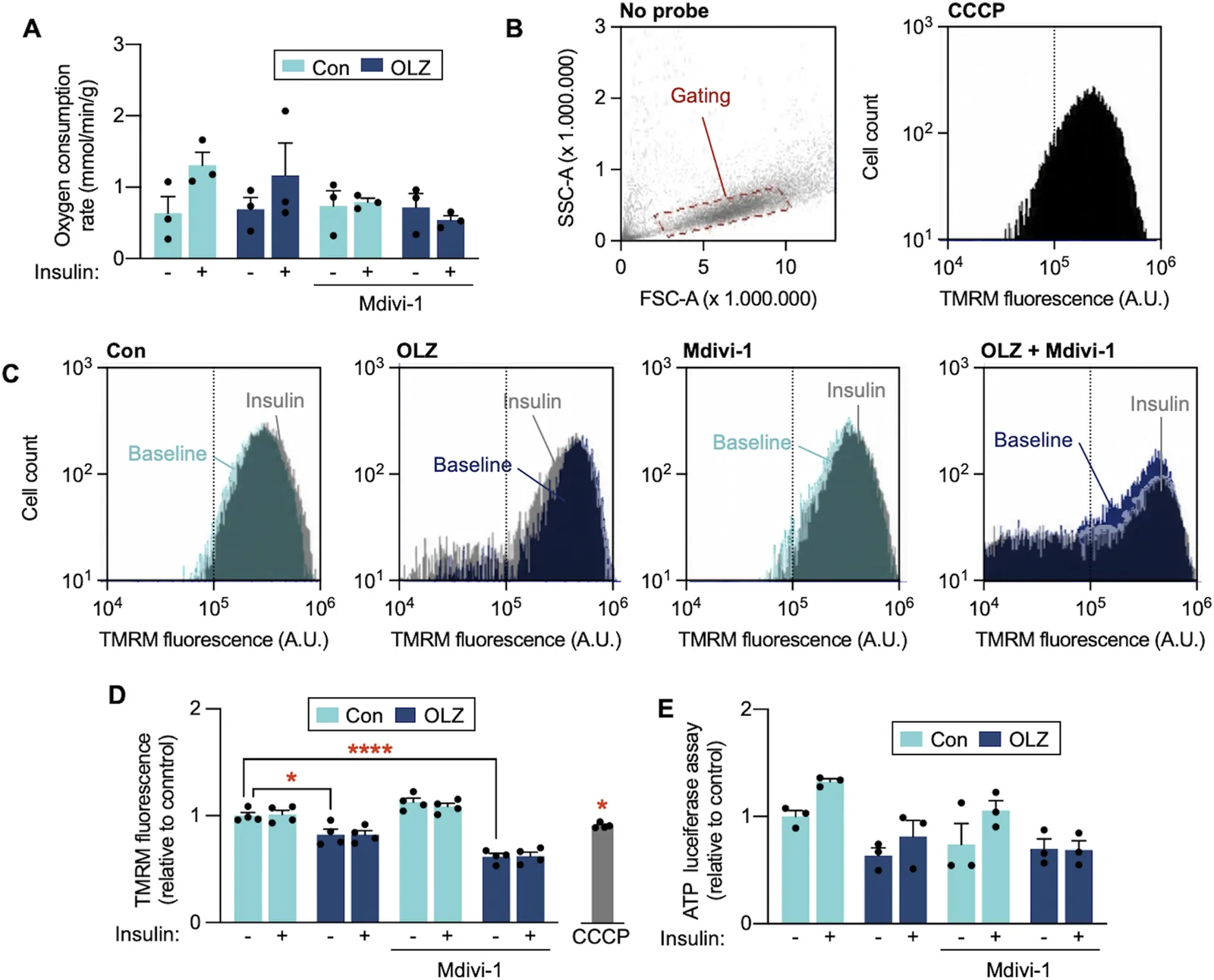
OLZ induced a slight decrease in mitochondrial bioenergetics in L6 muscle cell line, which is not rescued by Mdivi-1. L6 myoblasts were incubated with 160 µM OLZ and/or 10 µM Mdivi-1 for 24 h, then subjected to a 30 min treatment with 100 nM insulin. **(A)** Resting oxygen consumption rate as a measure of mitochondrial respiration, assessed through oxygraphy (n = 3). **(B)** Left: representative side-forward scatter dot plot used for initial gating in flow cytometry. Right: representative histogram of TMRM intracellular fluorescence of cells treated with 50 µM CCCP for 30 min as a positive control for decreased mitochondrial potential. **(C)** Representative histograms of TMRM intracellular fluorescence of untreated (turquoise/navy blue) or insulin-treated (gray shadow) cells. **(D)** Quantification of the mitochondrial transmembrane potential assessed as in panel **(C)** (n = 4). **(E)** Intracellular ATP levels evaluated using a bioluminescent assay (n = 3). Con: control, OLZ: olanzapine. Results are shown as mean ± SEM. Data of the oxygen consumption rate and mitochondrial transmembrane potential analyses **(A,B)** passed the normality test and were analyzed via two-way ANOVA followed by a Bonferroni post-test. Since the data of the ATP levels analysis **(C)** did not pass the Shapiro-Wilk normality test, groups’ means were compared using the Kruskal-Wallis test followed by a Dunn’s test. *p < 0.05 and ****p < 0.0001.

## 3 Results

### 3.1 Mdivi-1 rescues OLZ-induced loss in cell viability in L6 myoblasts

Since skeletal muscle may be particularly affected by SGAs through mitochondrial fragmentation, we used an L6 myoblasts model to evaluate the metabolic effects of OLZ and their potential rescue by Mdivi-1. First, we assessed overall cell morphology through bright-field microscopy (Figure 1A). Images showed that OLZ alone does not alter cell morphology, while Mdivi-1 *per se* apparently increases cell size. Interestingly, the combination of both drugs prevented the cytomegaly, but induced cell vacuolization. Then, we evaluated cell viability through a propidium iodide (PI) incorporation assay via flow cytometry (Figures 1B–D). We used a wide gating strategy that encompassed the main cellular population regardless of the changes in cell size and granularity (Figure 1B). Our results show that treatment with OLZ caused a slight but significant decrease in cell viability (x̅ = 91%, p = 0.023), whereas 1 µm Mdivi-1 (x̅ = 96.17%) and Mdivi-1 10 µm (x̅ = 95.33%) maintained similar cell viability values relative to control condition (Figures 1C,D). The combined OLZ and 10 µM Mdivi-1 treatment exhibited average cell viability values without statistical significance relative to control condition, which suggests that, at that concentration, Mdivi-1 exerts a protective effect that rescues L6 myoblasts from OLZ-induced cell death. In turn, 1 µM Mdivi-1 did not exert such protection. Therefore, the 10 µM concentration was selected for subsequent experiments. Interestingly, we observed a distribution displacement to the left in the cells treated with 10 µM Mdivi-1 either with or without OLZ (Figure 1C, right panels), which may be associated with the increased size or granularity of the cells. However, since it was only observed in viable cells that do not significantly incorporate PI, it could be due to changes in autofluorescence.

### 3.2 Mdivi-1 does not rescue OLZ-mediated disruption of insulin signaling in L6 myoblasts

After corroborating that Mdivi-1 reduced OLZ-induced cell death, we tested if the role of Mdivi-1 on OLZ-induced insulin signaling disruption in our model. As expected, a 30 min treatment with 100 nM insulin induced significant increase in the phosphorylation state of Akt at Ser^473^ and of Erk1/2 at Thr^202^/Tyr^204^ (Figure 2), along with a significant increase in glucose uptake (Figure 4). As previously reported, pre-incubation with OLZ promoted the disruption of the insulin signaling axis, since insulin stimulation did not produce statistically significant changes in Akt and ERK1/2 phosphorylation. Consequently, glucose uptake failed to increase in the OLZ-incubated condition. Mdivi-1 pre-treatment alone, in turn, abrogated both Akt and ERK phosphorylation, without affecting glucose uptake. Of note, even when Mdivi-1 protected from the OLZ-induced decrease in cell viability, pre-treatment with Mdivi-1 failed to prevent the OLZ-induced loss in the insulin response both in the signaling (Figure 2) and functional (Figure 4) parameters.

**FIGURE 4:**
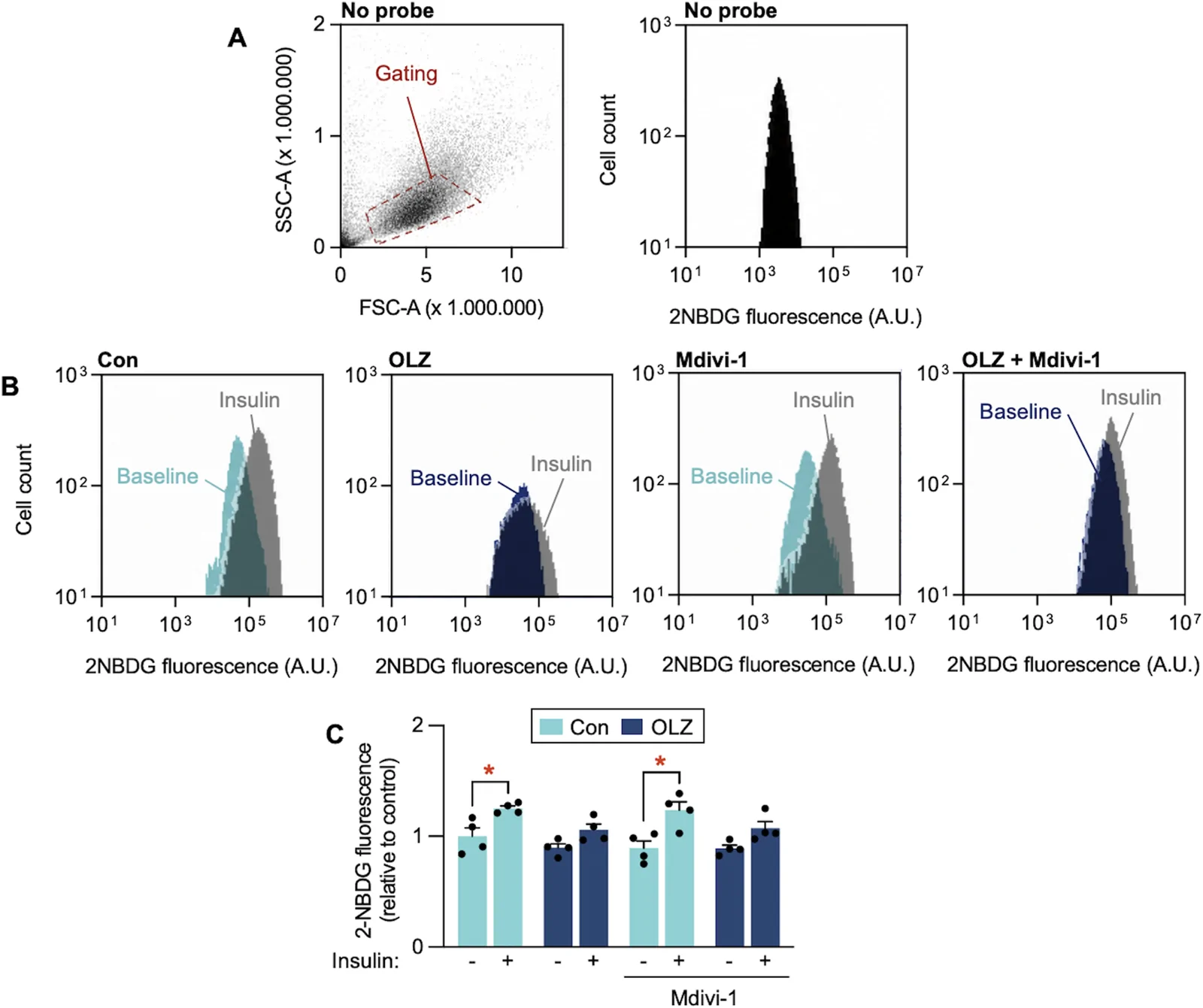
OLZ disrupts insulin-induced glucose uptake in the L6 muscle cell line, which is not rescued by Mdivi-1 treatment. L6 myoblasts were incubated with 160 µM OLZ and/or 10 µM Mdivi-1 for 24 h, then subjected to a 30 min treatment with 100 nM insulin. **(A)** Left: representative side-forward scatter dot plot used for initial gating in flow cytometry. Right: representative histogram of baseline fluorescence of cells without probe. **(B)** Representative histograms of 2NBDG intracellular fluorescence of untreated (turquoise/navy blue) or insulin-treated (gray shadow) cells. **(C)** Quantification of the glucose uptake in L6 cells evaluated as in panel B (n = 4). Con: control, OLZ: olanzapine. Results are shown as mean ± SEM. Since the data passed the Shapiro-Wilk normality test they were analyzed via two-way ANOVA followed by a Bonferroni post-test. *p < 0.05.

### 3.3 Mdivi-1 does not rescue OLZ-mediated disruption of mitochondrial bioenergetics in L6 myoblasts

Then, we evaluated the effect of OLZ and Mdivi-1 on insulin-mediated changes in mitochondrial bioenergetics. Under our experimental conditions, insulin did not significantly change oxygen consumption, mitochondrial membrane potential or intracellular ATP content compared to the untreated cells (Figure 3). Interestingly, and similar to our results on insulin-mediated glucose uptake, OLZ pre-treatment either with or without Mdivi-1 decreased baseline mitochondrial potential (Figure 3D). In summary, the cellular capacity to maintain the insulin-induced glucose uptake response could be apparently dependent on the intact mitochondrial potential.

### 3.4 OLZ induced mitochondrial fragmentation in L6 myoblasts, while combination with Mdivi-1 presented mixed effects

Since we did not observe any changes on the mitochondrial metabolic state with our treatments, we evaluated the impact of OLZ and Mdivi-1 on mitochondrial morphological parameters to check if, indeed, Mdivi-1 rescues a potential OLZ-mediated mitochondrial fragmentation (Figure 5A). As reported in the literature, OLZ caused mitochondrial fragmentation, which was observed as a significant increase in the number of fragmented mitochondria (p < 0.0001) (Figure 5B), accompanied by a significant decrease in the number of mitochondrial networks (p < 0.001) (Figure 5C). On the other hand, Mdivi-1 was expected to promote inhibition of mitochondrial fragmentation, which should translate into an elongated mitochondrial phenotype; however, those changes were not statistically observed (Figure 5D), which could suggest a high baseline level of mitochondrial fusion in L6 cell model. Furthermore, when both compounds were combined, Mdivi-1 partially rescued the fragmentation of the mitochondrial network caused by OLZ (Figure 5C), but surprisingly, caused a decrease in elongated mitochondria (Figure 5D). Finally, the total number of mitochondria remained similar among the groups, suggesting that the observed changes were not caused by changes in mitochondrial biomass, i.e., biogenesis or mitophagy, but mainly due to changes in its morphology.

**FIGURE 5:**
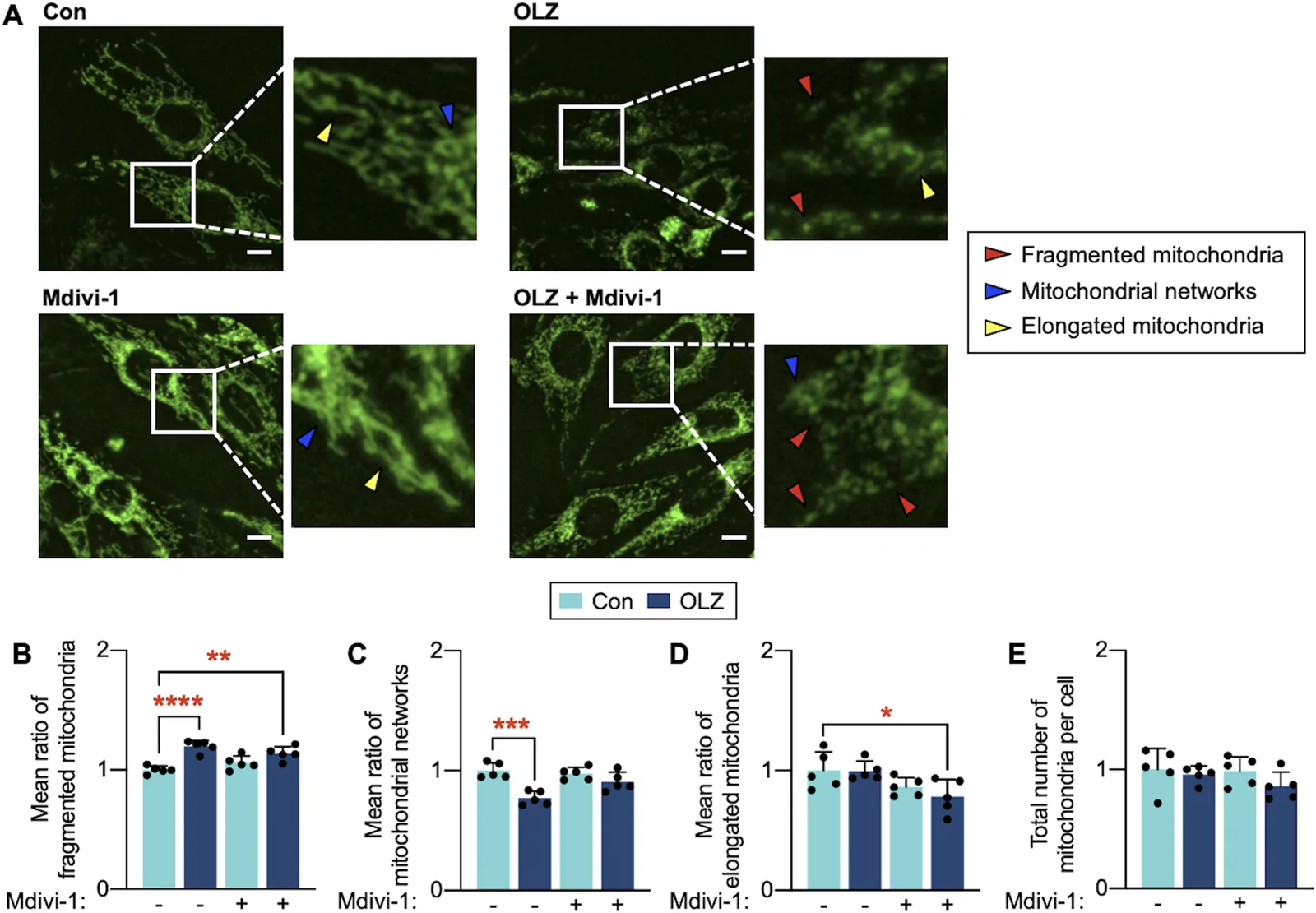
OLZ induces mitochondrial fragmentation in the L6 muscle cell line, while combination with Mdivi-1 presented mixed effects. **(A)** Representative images of Mitotracker Green-loaded L6 myoblasts, assessed through fluorescence microscopy (n = 5). Red arrowheads: fragmented mitochondria (bullets). Blue arrowheads: elongated mitochondria (spaghetti). Yellow arrowheads: mitochondrial networks (branched). **(B)** Mean ratio of fragmented mitochondria (relative to control), evaluated as in **(A). (C)** Mean ratio of mitochondrial networks (relative to control), evaluated as in **(A). (D)** Mean ratio of elongated mitochondria (relative to control), evaluated as in **(A). (E)** Total number of mitochondria per cell (relative to control), evaluated as in **(A)**. Con: control, OLZ: olanzapine. Results are shown as mean ± SEM. Since the data passed the Shapiro-Wilk normality test, they were analyzed via two-way ANOVA followed by a Bonferroni post-test. *p < 0.05, **p < 0.01, ***p < 0.001 and ****p < 0.0001. Bar: 10 µm.

### 3.5 OLZ binds to the GTPase domain of Drp1 with similar affinity as Mdivi-1 and other Drp1 inhibitors assessed *in silico*

As Mdivi-1 did not fully protect from mitochondrial morphology changes and provided mixed effects in combination with OLZ, we probed whether OLZ might engage the mitochondrial fission machinery directly, and not only as an upstream inducer of fragmentation. For this, we modelled the interaction of OLZ with Drp1, the GTPase that executes mitochondrial fission and whose pharmacological inhibition with Mdivi-1 we had used throughout this study. Using the X-ray structure of the human Drp1 GTPase domain (residues 1–320; PDB 4H1V) with GMP-PNP and Mg^2+^ present in the catalytic site, we performed rigid-exhaustive docking (FRED, ChemGauss4 scoring) of OLZ alongside three structurally unrelated Drp1 GTPase inhibitors: Mdivi-1, Drpitor1a (Wu et al., 2020) and DRP1i27 (Rosdah et al., 2022). This panel allowed us to benchmark OLZ against *bona fide* Drp1 ligands within an identical computational pipeline.

All four compounds were predicted to dock within the same region of the protein, occupying the guanine-nucleotide (GTP-binding) pocket of the GTPase domain and overlapping the footprint of the native GTP cofactor (Figure 6). The docked GTP reference was consistent with the expected, extensive contact network of the nucleotide, including thirteen hydrogen bonds engaging the phosphate-binding P-loop and the G4/G5 motifs (Thr^33^, Gln^34^, Ser^36^, Gly^37^, Lys^38^, Ser^39^, Ser^40^, Gly^149^, Lys^216^, Asn^246^ and Gln^249^), a π-cation contact with Lys^216^, and salt bridges from the triphosphate moiety to Lys^38^ and from the guanine base to Asp^218^ (Figure 6B). Docking recovered pocket residues corresponding to catalytic determinants previously identified by *in silico* alanine-scanning mutagenesis identified as essential for Drp1 GTP hydrolysis (Lys^38^, Ser^39^, Ser^40^, Asp^146^, Gly^149^, Lys^216^, Asp^218^ and Asn^246^) (Wenger et al., 2013a), supporting the assignment of the modelled site as the functional nucleotide pocket and suggesting that the predicted poses are compatible with GTP-competitive binding. Within this shared pocket, docking and PRODIGY scoring of the top-ranked poses yielded comparable predicted binding free energies for the four ligands, clustered within ∼0.15 kcal·mol^-1^ of one another. OLZ returned the most favorable predicted binding affinity (ΔG = −5.11 kcal·mol^-1^), marginally ahead of DRP1i27 (ΔG = −5.08), Drpitor1a (ΔG = −4.97) and Mdivi-1 (ΔG = −4.96) (Figures 6C–F).

**FIGURE 6:**
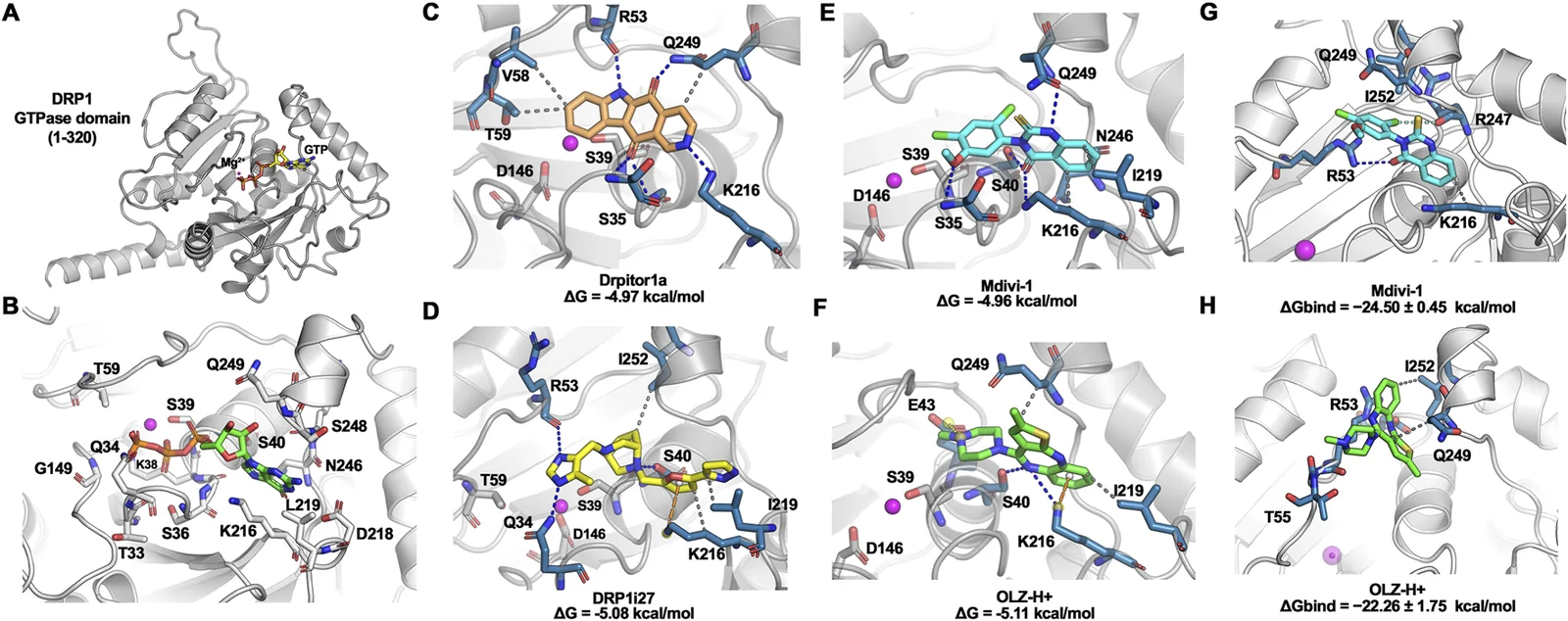
Molecular Docking of Drp1 inhibitors and Molecular Dynamics pose relaxation with MM/PBSA affinity calculations. Molecular models of the human DRP1 GTPase domain (PDB ID: 4H1V) bound to catalytic ${\text{Mg}}^{2+}$ were evaluated for small-molecule binding. **(A)** Crystallographic fold showing the nucleotide cleft. **(B)** Co-crystallized GMP-PNP/ ${\text{Mg}}^{2+}$ reference pose. **(C–F)** Representative FRED docked poses for Drpitor1a, DRP1i27, Mdivi-1, and OLZ. ΔG values are PRODIGY scores (relative predicted binding energies, not experimental affinities). **(G,H)** MD cluster centroid binding modes: **(G)** Mdivi-1 (dominant cluster occupancy = 98.3%, cyan sticks) and **(H)** protonated olanzapine (OLZ-H^+^, dominant cluster occupancy = 39.8%, green sticks) superposed within the DRP1 catalytic pocket, both anchored by hydrophobic interactions with Ile252 (blue sticks). Catalytic Mg^2+^ (magenta sphere) dissociates into bulk solvent in both inhibitor complexes (occupancy = 0.000). ΔGbind values are MM/PBSA calculations at ε_in_ = 4.0. Residue numbering follows the human Drp1 GTPase-domain construct according to Wenger et al., 2013b. OLZ: olanzapine.

### 3.6 Molecular dynamics and MM/PBSA profiling reveal decisive protonation-dependent pocket retention and comparable affinity between Mdivi-1 and Olanzapine-H^+^

Because static molecular docking scores reflect rigid crystal lattices and cannot capture solvent displacement, dynamic pocket breathing, or conformational drift, we subjected the docked complexes to 10.0 ns of unrestrained all-atom molecular dynamics simulations and 200-snapshot MM/PBSA continuum solvation thermodynamic profiling across the standardized 4H1V DRP1/Mg^2+^ receptor framework (Table 1).

**TABLE 1:** Conformational stability metrics, catalytic Mg^2+^ coordination, and end-state MM/PBSA binding free energies across molecular dynamics trajectories for human DRP1 complexes.

Ligand	Formal Charge ( q )	C α RMSD (Å) [95% CI]^a^	Pocket Backbone RMSD (Å)^b^	Ligand Pose RMSD (Å)^c^	Ligand COM Drift (Å)^d^	Ligand Buried Fraction^e^	Dominant Cluster Occupancy^f^	${\text{Mg}}^{2+}$ Ser39:OG Occupancy^g^	$\Delta G_{\text{bind}}$ ( $\epsilon_{\text{in}}=2.0$ ) (kcal/mol)^h^	$\Delta G_{\text{bind}}$ ( $\epsilon_{\text{in}}=4.0$ ) (kcal/mol)^h^
GTP	−4	1.95 [1.67, 2.22]	1.18 ± 0.15	1.58 ± 0.26	1.16 ± 0.30	0.85 ± 0.02	100.0% (1 cluster)	0.999	−127.41±4.86	−90.73±3.18
DRP1i27	+1	2.03 [1.84, 2.23]	1.62 ± 0.23	8.16 ± 17.73	5.51 ± 18.58	0.60 ± 0.14	39.2% (32 clusters)	0.000	−24.33±1.35	−28.91±1.41
Mdivi-1	0	2.70 [2.30, 3.10]	1.15 ± 0.13	4.00 ± 0.58	0.88 ± 0.40	0.61 ± 0.04	98.3% (6 clusters)	0.000	−19.14±0.46	−24.50±0.45
Olanzapine (OLZ-H^+^)	+1	2.94 [2.37, 3.50]	1.65 ± 0.32	7.94 ± 1.56	4.16 ± 1.69	0.47 ± 0.10	39.8% (51 clusters)	0.000	−19.18±1.55	−22.26±1.75
Drpitor1a	0	2.27 [1.73, 2.82]	1.52 ± 0.23	8.44 ± 1.61	5.35 ± 1.88	0.52 ± 0.11	35.6% (46 clusters)	0.000	−15.86±1.05	−19.18±1.46
Olanzapine (OLZ neutral)	0	2.19 [2.02, 2.36]	1.79 ± 0.29	16.01 ± 14.07	12.69 ± 15.72	0.22 ± 0.12	7.8% (283 clusters)	0.000	−9.06±1.61	−10.41±1.90

Data are expressed as mean ± SD, across 1,000 frames unless indicated otherwise.

^*a*^ C α root-mean-square deviation (RMSD) relative to crystallographic reference (PDB 4H1V), with 95% confidence intervals corrected for autocorrelation time using Geyer initial-positive-sequence truncation ( $N_{\text{eff}}=N/\tau$ ).

^*b*^ Backbone RMSD, evaluated across the 23-residue canonical GTP-binding pocket (P-loop, NKxD, and G5 motifs: residues 33–40, 58–59, 148–149, 215–219, 244–249).

^*c*^ Ligand heavy-atom pose RMSD, computed in the protein-superposed reference frame.

^*d*^ Center-of-mass (COM) drift of the ligand relative to its initial docked coordinates.

^*e*^ Fraction of ligand solvent-accessible surface area buried by receptor heavy atoms, computed via Shrake–Rupley algorithm.

^f^ Conformational clustering using the GROMOS, algorithm at a 2.0 Å heavy-atom RMSD, cutoff across all 1,000 production frames.

^g^ Fraction of trajectory frames in which catalytic ${\text{Mg}}^{2+}$ maintains inner-shell coordination (d ≤ 2.6Å) to Ser39:OG.

^h^ End-state MM/PBSA, effective binding free energy evaluated across 200 snapshots (50-ps stride over 10.0 ns) at physiological ionic strength (0.15 M NaCl) with solute interior dielectric (ϵ_in_ = 2.0) or 4.0 (ϵ_ext_ = 80.0). Standard errors of the mean are corrected for autocorrelation using automated Sokal windowing (*N*_eff_ = *N*/2τ_int_) and cross-validated with Flyvbjerg–Petersen blocking. The GTP, control carries a net −4, charge and retains Mg2+, serving as a positive reference.

Analysis of trajectory dynamics over the full 10.0-ns ensemble demonstrated that ligand ionization state is the decisive determinant governing olanzapine pocket retention. Unprotonated neutral olanzapine (OLZ) exhibited marked instability within the nucleotide cleft: its center-of-mass (COM) drifted 12.69 ± 15.72 Å away from the initial binding site (reaching a final separation of 61.70 Å), its pocket backbone RMSD relaxed to 1.79 ± 0.29 Å, and its buried surface fraction collapsed to 0.22 ± 0.12 (terminating at 0.00 at 10.0 ns). Conformational clustering via the GROMOS algorithm (2.0 Å cutoff) resolved 283 distinct clusters with the dominant pose accounting for only 7.8% of trajectory frames, corresponding to near-complete unbinding and pocket egress (contacting canonical pocket residues in only 6.9% of frames). In stark contrast, piperazine-protonated cationic olanzapine (OLZ-H^+^) remained stably anchored within the catalytic cleft, displaying a COM drift of only 4.16 ± 1.69 Å, a ligand pose RMSD of 7.94 ± 1.56 Å, a buried surface fraction of 0.47 ± 0.10, and pocket retention in 57.1% of frames across 51 clusters (dominant pose occupancy = 39.8%). Mdivi-1 exhibited high pose fidelity, maintaining a single dominant conformational cluster (98.3% occupancy) with a COM drift of 0.88 ± 0.40 Å and pose RMSD of 4.00 ± 0.58 Å (Table 1). Tracking catalytic Mg^2+^ coordination persistence revealed that while Mg^2+^ remained chelated to Ser39:OG in the native GTP control (mean distance 2.14 Å, occupancy = 0.999), the ion dissociated into bulk solution in all five inhibitor complexes (mean distances 4.23–4.74 Å, occupancy = 0.000, 4.0–4.6 hydrating waters), confirming that small-molecule inhibitors do not engage the catalytic divalent cation.

End-state MM/PBSA binding free energy calculations across 200 snapshots spanning the entire 10.0-ns trajectory demonstrated that protonated olanzapine (OLZ-H^+^) and Mdivi-1 exhibit statistically indistinguishable binding affinities for the DRP1 catalytic domain at both solute interior dielectrics (Table 1). At ε_in_ = 4.0, the effective binding free energy of Mdivi-1 was ΔG_bind_ = −24.50 ± 0.45 kcal/mol compared to −22.26 ± 1.75 kcal/mol for OLZ-H^+^ (pairwise contrast ΔΔG = −2.24 ± 1.80 kcal/mol; Welch’s t = −1.24, df = 7.6, p = 0.252, Benjamini–Hochberg FDR q = 0.252). At ε_in_ = 2.0, Mdivi-1 (ΔG_bind_ = −19.14 ± 0.46 kcal/mol) and OLZ-H^+^ (ΔG_bind_ = −19.18 ± 1.55 kcal/mol) were virtually identical (ΔΔG = +0.04 ± 1.62 kcal/mol; Welch’s t = 0.02, df = 7.6, p = 0.982, FDR q = 0.982). Both compounds share a critical, persistent hydrophobic contact with Ile252 with occupancies of 0.899 for Mdivi-1 and 0.914 for OLZ-H^+^ (Figures 6G,H). Conversely, neutral olanzapine displayed severely compromised binding (ΔG_bind_ = −10.41 ± 1.90 kcal/mol at ε_in_ = 4.0), demonstrating that piperazine protonation provides a decisive −11.85 ± 2.58 kcal/mol stabilization (Welch’s t = 4.59, df = 15.4, p = 0.0003, FDR q = 0.00077) required for pocket engagement. The reference inhibitor DRP1i27 bound with the highest small-molecule affinity (−28.91 ± 1.41 kcal/mol at ε_in_ = 4.0), while native GTP yielded −90.73 ± 3.18 kcal/mol. These dynamic and thermodynamic calculations substantiate that protonated olanzapine possesses pocket affinity fully comparable to Mdivi-1, providing a physical basis for direct pocket competition while establishing that unprotonated olanzapine cannot maintain stable target engagement. Full MD methods, analysis and additional calculations involving the several interactions with relevant DRP1 ligands, and corresponding references are available in Supplementary Information.

## 4 Discussion

### 4.1 Principal findings

The clinical utility of SGAs like OLZ is frequently undermined by severe metabolic side effects, including weight gain and IR (Chen et al., 2017). While the association between mitochondrial dysfunction and impaired glucose homeostasis is well-documented in metabolic diseases (Koliaki and Roden, 2016), the specific role of mitochondrial dynamics in OLZ-induced IR remains poorly understood. In this study, we showed that OLZ induces significant mitochondrial fragmentation and impairs insulin signaling and glucose uptake in L6 skeletal muscle cells. However, contrary to our initial hypothesis, pharmacological inhibition of mitochondrial fission via Mdivi-1 was insufficient to rescue the insulin-resistant phenotype. Regarding our selection of OLZ working concentration, a systematic review published in 2023 showed that the therapeutic blood OLZ concentrations typically range from 20 to 40 ng/mL, corresponding to approximately 0.064–0.128 μM (Wesner et al., 2023). Although this range is substantially lower than the concentration used in the present study (160 μM), this discrepancy largely reflects the high plasma protein binding of olanzapine (approximately 93% *in vivo*) (Goodman and Gilman, 2001), as well as the lower sensitivity of immortalized cell lines compared with primary cells.

A primary finding of this study is the dissociation between mitochondrial network structure and metabolic function under OLZ treatment. It is widely accepted that mitochondrial dysfunction and disrupted insulin signaling are highly associated in the pathogenesis of metabolic disorders (Pinti et al., 2019; Sergi et al., 2019). Previous literature suggests that preventing mitochondrial fragmentation can restore insulin signaling in various models of Type 2 Diabetes (Chiu et al., 2021; Finocchietto et al., 2022). Our results show that Mdivi-1 has mixed effects on the OLZ-triggered mitochondrial fragmentation: it promotes a moderate preservation of the mitochondrial networks but fails to rescue mitochondrial fragmentation. Furthermore, the OLZ-Mdivi-1 combination fragments elongated mitochondria. Concomitantly, Mdivi-1 failed to prevent the detrimental effects of OLZ on Akt and ERK phosphorylation or glucose uptake. These suggest that OLZ-induced mitochondrial fragmentation occurs concurrently with loss in insulin responsiveness, which is Mdivi-1 resistant. Interestingly, these results in mitochondrial fragmentation correlate with the cell vacuolization observed using bright-field microscopy upon OLZ treatment (Figure 1A). However, the distribution displacement upon Mdivi-1 treatment either with or without OLZ remains unexplained (Figure 1C). Whether this observation is associated with our proposed mechanism is unclear; however, based on our current data, exploring this parameter is unlikely to significantly alter the conclusions of the present work.

In agreement with previous reports, insulin stimulation successfully increased Akt and ERK phosphorylation and enhanced glucose uptake in L6 cells (Wang et al., 2018), confirming the integrity of the insulin-responsive signaling pathway in our model. OLZ treatment markedly impaired this response, reducing both kinase phosphorylation and glucose uptake, consistent with an insulin-resistant phenotype. The combined OLZ-Mdivi-1 treatment failed to restore the insulin response, as cells displayed a pattern similar to OLZ treatment alone.

We further observed that OLZ decreased the mitochondrial transmembrane potential, an effect that was not prevented by Mdivi-1 (Figure 3). Of note, Mdivi-1 reportedly interferes with mitochondrial bioenergetics by inhibiting Complex I of the mitochondrial electron transport chain, leading to an imbalance in mitochondrial ROS production (Bordt et al., 2017; Aishwarya et al., 2020). Thus, this effect summed with OLZ toxicity may account for the failed Mdivi-1-mediated rescue.

Thus, this observation raises three possibilities: (a) Mdivi-1 may possess inherent inhibitory properties on mitochondrial respiration in L6 cells, which could mask any potential metabolic benefits of preventing fission. This is reinforced by our observation that Mdivi-1 treatment alone decreased insulin-induced Akt and ERK phosphorylation (Figure 2). (b) The deleterious effects of OLZ likely stem from a direct interference with the insulin signaling cascade that cannot be bypassed by Mdivi-1. This notion is supported by our previous work that shows that OLZ directly binds to the extracellular domain of the insulin (del Campo et al., 2018). (c) OLZ hinders Mdivi-1’s effect by directly affecting its mechanism of action. This idea is supported by our novel observation that OLZ binds to the GTP-binding domain of Drp1 (Figure 6).

Docking OLZ into the GTPase domain of human Drp1 (Wenger et al., 2013a), in parallel with three established Drp1 inhibitors, placed OLZ squarely within the guanine-nucleotide pocket, overlapping the GTP footprint and recruiting the catalytic anchors Ser^40^, Lys^216^ and Gln^249^ that are required for Drp1 GTP hydrolysis (Wenger et al., 2013b). Its predicted binding score (−5.11 kcal·mol^-1^) was comparable to, and marginally more favorable than, those of Mdivi-1, Drpitor1a (Wu et al., 2020) and DRP1i27 (Rosdah et al., 2022). Notably, the GTPase domain is the most highly conserved module of the dynamin superfamily, in which an N/TKxD (G4) motif built around Lys^216^/Asp^218^ confers guanine-nucleotide specificity (Niemann et al., 2001); the recovery of these residues supports the assignment of the predicted binding pose to the GTP-binding pocket and is consistent with a potential GTP-competitive binding mode. This raises the possibility of a structural basis for the long-standing observation that OLZ perturbs mitochondrial dynamics (del Campo et al., 2018): beyond indirect, signaling-driven effects, OLZ has the physicochemical features–an aromatic tricyclic core for a Lys^216^ π-cation contact and a basic piperazine nitrogen for a Glu^43^ salt bridge–needed to occupy the catalytic pocket of the fission GTPase itself.

This structural layer must, however, be reconciled with the biology rather than over-interpreted, and it in fact reinforces our central conclusion. If OLZ behaved as a classical GTP-competitive Drp1 inhibitor, occupancy of the nucleotide pocket would be expected to suppress fission; yet OLZ biologically promotes mitochondrial fragmentation (Figure 5) (del Campo et al., 2018). Predicted pocket occupancy therefore does not translate straightforwardly into functional GTPase inhibition, as ligand binding does not necessarily induce the conformational changes required for enzymatic inhibition. Indeed, although OLZ shares several catalytic contacts with established Drp1 inhibitors, its predicted interaction profile also includes distinct electrostatic contacts that may reflect a different binding geometry and, consequently, a different functional outcome. This interpretation is further supported by the narrow ∼5 kcal·mol^-1^ band into which all four ligands fell, including Mdivi-1, whose direct activity against human Drp1 has been questioned and attributed in part to weak, possibly aggregation-prone and off-target engagement (Rosdah et al., 2022).

A central mechanistic question arising from our findings is why pharmacological inhibition of DRP1 with Mdivi-1 rescued cell viability but failed to reverse olanzapine-mediated insulin resistance and mitochondrial fragmentation. In our initial *in silico* docking screen, olanzapine docked into the DRP1 catalytic cleft with scores comparable to Mdivi-1 (−5.11 vs. −4.96 kcal/mol), suggesting potential competitive antagonism. Our 10.0-ns all-atom molecular dynamics simulations and 200-snapshot MM/PBSA continuum thermodynamic profiling provide critical physical clarification of this interaction. First, MD simulations revealed that olanzapine pocket retention is strictly dependent on the protonation state of its piperazine nitrogen. Neutral olanzapine is rapidly displaced from the pocket (mean COM drift of 12.69 Å, buried surface fraction collapsing to zero, and pocket contact in only 6.9% of frames), yielding an unfavorable binding energy of −10.41 ± 1.90 kcal/mol (ε_in = 4.0). In contrast, the physiologically predominant protonated cation (OLZ-H^+^, pKa ∼7.8) is stably accommodated within the catalytic pocket (COM drift = 4.16 ± 1.69 Å, 57.1% pocket occupancy), gaining an energetic advantage of −11.85 ± 2.58 kcal/mol (p = 0.0003, FDR q = 0.00077) driven by strong electrostatic pairing with acidic pocket residues Glu43 (−10.8 kcal/mol) and Asp49 (−10.1 kcal/mol). Second, thermodynamic contrast testing over the complete 10-ns sampling window demonstrated that Mdivi-1 and Olanzapine-H^+^ exhibit statistically indistinguishable binding affinities for DRP1 (ΔΔG = −2.24 ± 1.80 kcal/mol, q = 0.252 at ε_in = 4.0; ΔΔG = +0.04 ± 1.62 kcal/mol, q = 0.982 at ε_in = 2.0). Both ligands converge upon and share a common hydrophobic anchor residue, Ile252 (occupancies of 0.899 for Mdivi-1 and 0.914 for OLZ-H^+^), while neither compound coordinates the catalytic Mg^2+^ ion. This establishes that OLZ-H^+^ and Mdivi-1 can mutually compete for occupancy of the DRP1 catalytic cleft without an insurmountable thermodynamic barrier favoring one over the other. However, this competitive comparability *in silico* underscores that DRP1 pocket binding alone cannot account for the persistence of mitochondrial fragmentation and insulin signaling collapse when both compounds are co-administered in muscle cells.

Although OLZ and Mdivi-1 displayed comparable predicted binding free energies, OLZ was used at a 16-fold higher concentration (160 µM versus 10 µM) to induce mitochondrial fragmentation and insulin resistance. This observation suggests that binding to the Drp1 GTPase domain alone is unlikely to account for the biological activity of OLZ under our experimental conditions. Instead, the substantially higher concentration required is more consistent with the contribution of additional molecular targets, in agreement with the well-established polypharmacology of OLZ. Moreover, given the pleiotropic pharmacology of OLZ, receptor-mediated pro-fission signaling may predominate over any potential direct interaction with the Drp1 GTPase domain. Read in this light, the docking analysis is compatible with our functional findings modeling: even though OLZ can contact Drp1, stabilizing a fused network with Mdivi-1 neither normalized insulin signaling nor restored bioenergetics. Together with the reported Drp1-independent actions of Mdivi-1, these findings suggest that modulation of mitochondrial fission alone is insufficient to restore insulin responsiveness in OLZ-treated cells, again pointing to extramitochondrial and Drp1-independent targets as potential contributors to OLZ-induced insulin resistance.

### 4.2 Alternative signaling mechanisms

It is important to note that the contribution of extramitochondrial signaling pathways may extend beyond direct interactions with the insulin receptor. L6 skeletal muscle cells express several metabolically relevant G protein-coupled receptors (GPCRs) whose activation can modulate cellular signaling and glucose metabolism. For example, endothelin-1 activates ERK1/2 in L6 myoblasts through transactivation of the platelet-derived grown factor receptor, a signaling pathway implicated in impaired muscle differentiation and insulin resistance (Harada et al., 2014). In addition, 5-HT2A receptors are expressed in L6 myotubes, and their activation stimulates glucose uptake by increasing plasma membrane GLUT1, GLUT3, and GLUT4 independently of IRS1, PI3K, and Akt signaling, demonstrating that receptor-mediated pathways can regulate glucose transport independently of the canonical insulin pathway (Hajduch et al., 1999). PAR-2 is also expressed and functional in L6 cells, where its activation stimulates myoblast proliferation through COX-2-dependent signaling (Duchesne et al., 2011). Similarly, GPR43 is expressed in differentiated L6 myotubes and can regulate intracellular Ca2+ signaling and downstream AMPK, PGC-1α, and muscle-related pathways (Maruta and Yamashita, 2020). Membrane-mediated androgen signaling has also been described in L6 cells, where testosterone promotes cellular responses through GPCR-dependent mechanisms involving PKC/ERK1/2 and PKA rather than the classical intracellular androgen receptor (Fu et al., 2012). Finally, opioid receptor activation has been shown to enhance glucose uptake in skeletal muscle cells through AMPK activation and GLUT4 translocation (Kairupan et al., 2019). Collectively, these findings highlight the presence of multiple extramitochondrial receptor-mediated pathways in L6 cells that can influence glucose metabolism and intracellular signaling. Given the pleiotropic pharmacology of OLZ, these pathways may represent potential contributors to its metabolic effects. In particular, 5-HT2A may be relevant to OLZ-induced metabolic effects, as OLZ has been shown to act as a potent antagonist of 5-HT2A-mediated signaling (Gaitonde et al., 2024), while 5-HT2A activation stimulates glucose uptake in L6 myotubes independently of the canonical IRS1/PI3K/Akt pathways (Hajduch et al., 1999).

The present findings are consistent with previous observations in clozapine-treated cells, where mitochondrial depolarization, increased ROS production, and mitochondrial fragmentation have also been described (Contreras-Shannon et al., 2013; Shao, 2022). Because clozapine and OLZ are closely related tricyclic SGAs sharing similar structural scaffolds and receptor-binding profiles, it is conceivable that they may perturb common mitochondrial pathways. In this context, our docking analysis raises the possibility that interaction with the Drp1 GTPase domain could represent one such mechanism. However, this hypothesis remains speculative and requires direct biochemical validation.

From a broader pharmacological standpoint, these findings should be viewed as hypothesis-generating and require biochemical validation. The analysis identifies the Drp1 GTPase pocket as one credible molecular contact point for OLZ and offers a structural framework that could help explain its reported impact on mitochondrial fission/fusion balance (del Campo et al., 2018), but it does not, on its own, establish that this interaction accounts for OLZ’s metabolic toxicity. Several limitations temper interpretation: The binding fingerprint of OLZ is sensitive to its protonation state, such that we relied on the neutral-form pose (Figure 6F) for the contact analysis; and a direct functional readout, a Drp1 GTPase or binding assay with OLZ, as performed for DRP1i27 by surface plasmon resonance and microscale thermophoresis (Rosdah et al., 2022), was beyond the scope of the present cellular study. Such experiments, together with complementary techniques such as co-immunoprecipitation and Drp1 oligomerization assays, represent an important next step to determine whether the predicted interaction between OLZ and Drp1 is functionally relevant. Future studies should also employ more selective Drp1 inhibitors, such as Drpitor1a and DRPi27, to better distinguish the contribution of Drp1-mediated mitochondrial fission from the reported Drp1-independent inhibition of mitochondrial Complex I by Mdivi-1. Ultimately, integrating these biochemical approaches with structure-guided medicinal chemistry will be necessary to determine whether OLZ-Drp1 interactions contribute directly to insulin resistance or merely accompany the broader signaling alterations induced by OLZ in skeletal muscle cells.

While previous studies have shown that other SGAs, such as clozapine, alter mitochondrial morphology and reduce ATP levels (Contreras-Shannon et al., 2013), our work highlights a critical nuance: the morphological state of the mitochondria does not always dictate the metabolic status of the cell. Our findings suggest that in the context of OLZ-induced IR, the metabolic dysfunction may be driven by direct inhibition of signaling nodes, such as the disruption of ERK phosphorylation, rather than being a secondary consequence of a fragmented mitochondrial network.

In conclusion, our study shows that while OLZ induces mitochondrial fragmentation, reduced viability, and insulin resistance in L6 myoblasts, these phenomena can be pharmacologically uncoupled. The inability of Mdivi-1 to restore insulin sensitivity and mitochondrial bioenergetics despite rescuing viability suggests that OLZ-induced insulin resistance is mediated by pathways independent of Drp1-mediated fission, a view further supported by molecular docking, which, although it places OLZ within the Drp1 GTP-binding pocket with an affinity comparable to established Drp1 inhibitors, cannot reconcile such occupancy with the drug’s pro-fission, insulin-desensitising phenotype. Future investigations should target the specific extramitochondrial mechanisms by which OLZ impairs the insulin signaling cascade to develop better co-therapies for patients requiring SGA treatment.

## Data Availability

The raw data supporting the conclusions of this article will be made available by the authors, without undue reservation.
