# Supplementary material for: Olanzapine induces Mdivi-1-resistant disruption of insulin signaling in skeletal muscle cells
Source: Front Pharmacol. 2026 Oct 26;17:1920978. doi: 10.3389/fphar.2026.1920978 (PMC13745686; doi:10.3389/fphar.2026.1920978)
Supplement: Supplementary file 1 [file Supplementaryfile1.docx]

SUPLEMMENTARY MATERIAL

Olanzapine induces Mdivi-1-resistant disruption of insulin signaling in skeletal muscle cells

Pablo Arias^1^*, Catalina Salamanca^1^*, Fernanda Briones-Manriquez^1^, Lucas Oyanader^1^, Andrea Marimán^1^, Gonzalo Almarza^1^, Edson Yañez^2^, Juan Rodriguez^2^, Rodrigo Troncoso^2^, Leonel Rojo^3,4^, Carlos F. Lagos^5,6^, Roberto Bravo-Sagua^7,8,9,#^ and Andrea del Campo^1,10,#^.

^1^Laboratorio de Fisiología y Bioenergética Celular (LABFBE), Facultad de Química y de Farmacia, Pontificia Universidad Católica de Chile, Santiago 7810000, Chile.

^2^Laboratorio de Investigación en Nutrición y Actividad Física (LABINAF), Instituto de Nutrición y Tecnología de los Alimentos (INTA), Universidad de Chile, Santiago 7830490, Chile.

^3^Departamento de Biología, Facultad de Química y Biología, Universidad de Santiago de Chile (USACH), Santiago 9170465, Chile.

^4^Centro de Biotecnología Acuícola, Universidad de Santiago de Chile (USACH), Santiago 9170465, Chile.

^5^Chemical Biology & Drug Discovery Lab, Escuela de Química y Farmacia, Facultad de Ciencias, Universidad San Sebastián, Campus Los Leones, Lota 2465 Providencia 7510157, Santiago, Chile.

^6^Centro Basal Ciencia & Vida, Fundación Ciencia & Vida, Av. del Valle Norte 725, Huechuraba 8580702, Santiago, Chile.

^7^Laboratorio de Obesidad y Metabolismo (OMEGA), Instituto de Nutrición y Tecnología de los Alimentos (INTA), Universidad de Chile, Santiago 7830490, Chile.

^8^Advanced Center for Chronic Diseases (ACCDiS), Universidad de Chile & Pontificia Universidad Católica de Chile, Santiago 8380492, Chile.

^9^Centro Interuniversitario de Envejecimiento Saludable (CIES), Consorcio de Universidades del Estado de Chile (CUECH), Santiago 8320216, Chile.

^10^International Research Center for Medical Sciences, Kumamoto University, Kumamoto, Japan.

*** Correspondence:**
Andrea del Campo
Av. Vicuña Mackenna 4860, Macul, Santiago 7810000, Chile
[andrea.delcampo@uc.cl](mailto:andrea.delcampo@uc.cl)

Roberto Bravo-Sagua
Av. El Líbano 5524, Macul, Santiago 7830490, Chile
[rbravosagua@inta.uchile.cl](mailto:rbravosagua@inta.uchile.cl)

**Supplementary Methods**

**S1. Unified receptor construction and ligand chemistry**

All six complexes were constructed on the same human DRP1 GTPase-domain receptor (chain A, residues 1-321) and catalytic Mg2+ coordinates taken from PDB 4H1V. The receptor heavy-atom coordinates were identical across all systems, so differences in the simulations arise from the ligand rather than receptor preparation. Formal ligand charges were -4 for GTP, 0 for Mdivi-1, neutral OLZ, and Drpitor1a, and +1 for protonated OLZ and DRP1i27. The protonated OLZ model carries the charge on the distal N-methyl piperazine nitrogen. Receptor protonation was assigned once at pH 7.4 using PROPKA3/PDB2PQR and then reused across the panel. Both neutral and protonated OLZ were simulated as independent systems without asserting a single numerical pKa.

**S2. Force-field parameterization and solvation**

The protein was modeled with AMBER ff14SB. Small molecules used GAFF2 with AM1-BCC partial charges and exact integer net-charge correction. GTP used the Meagher-Redman-Carlson polyphosphate parameters and Mg2+ the Li-Merz 12-6 parameters for TIP3P. Each complex was solvated in a truncated-octahedral TIP3P box with a 12.0-Å minimum buffer, neutralized, and brought to approximately 0.15 M NaCl with Joung-Cheatham ion parameters. Final solvated systems contained 63,276-64,057 atoms and no severe nonbonded overlaps.

**S3. Minimization and equilibration**

Systems underwent steepest-descent minimization followed by restrained NVT equilibration at 310.15 K and NPT equilibration at 1 bar. All six systems met the predefined solute force criterion and the NVT/NPT quality-control checks. An additional 0.5-ns unrestrained NPT pre-equilibration preceded production and was excluded from the analyzed trajectories. Two systems (Drpitor1a and DRP1i27) did not meet the separate pre-equilibration settling criterion in the early production-summary audit; this residual uncertainty is retained as a limitation rather than treated as a failed trajectory.

**S4. Production molecular dynamics**

Production simulations were run with OpenMM on CUDA/Tesla T4 hardware. Each system comprised 20 consecutive 0.5-ns segments (10.0 ns per complex; 60.0 ns aggregate) with coordinates written every 10 ps. PME electrostatics used a 1.0-nm real-space cutoff and 5×10-4 Ewald error tolerance. Bonds to hydrogen were constrained and TIP3P water was rigid. Production used a 4-fs timestep with hydrogen-mass repartitioning rather than the 2-fs timestep originally specified in the analysis plan; no 2-fs control was performed. The complete 1,000-frame trajectories were corrected for periodic-boundary imaging and aligned on the protein backbone.

**S5. Structural, clustering, contact, and Mg2+ analyses**

Global Cα RMSD, pocket-backbone RMSD, ligand pose RMSD, ligand internal RMSD, center-of-mass drift, per-residue RMSF, ligand solvent-accessible surface area, and buried fraction were calculated over all 1,000 frames. Pocket-backbone RMSD used the 23-residue GTP footprint (33-40, 58, 59, 148, 149, 215-219, 244-249), whereas pocket occupancy used a 30-residue P-loop/G-motif set (32-44, 60-64, 146-149, 205-207, 236-240) at a 4.5-Å contact cutoff. Hydrogen bonds used d≤3.5 Å and angle≥150°, hydrophobic contacts 4.5 Å, charged contacts 4.0 Å, and Mg2+ inner-shell coordination 2.6 Å. Ligand-heavy-atom GROMOS clustering used 2.0 Å in the receptor Cα frame, with 1.5- and 2.5-Å sensitivity checks. Periodic-image artifacts were diagnosed by comparing raw and minimum-image ligand-receptor distances frame by frame.

**S6. MM/PBSA and uncertainty analysis**

Single-trajectory MM/PBSA was run with MMPBSA.py on 200 snapshots per complex sampled every 50 ps from 0.05 to 10.0 ns. Calculations used the internal PBSA solver, 0.15 M ionic strength, solvent dielectric 80, mbondi2 radii, surface-area nonpolar solvation (inp=1), and εin values of 2 and 4. Mg2+ was retained in the receptor for the GTP control and stripped with solvent for the five inhibitors because only GTP retained direct inner-shell Mg2+ coordination. No entropy term was calculated. Integrated autocorrelation times were estimated by Sokal automatic windowing and used to obtain effective sample sizes and corrected standard errors; Flyvbjerg-Petersen blocking provided an independent uncertainty cross-check. Pairwise inhibitor contrasts used Welch-Satterthwaite tests with Benjamini-Hochberg correction. Per-residue decomposition (idecomp=1) was performed at εin=4 over the same 200 snapshots in five equal shards; a mean contribution ≤-1.0 kcal mol-1 was used only as a reporting threshold, not a significance test.

**Supplementary Tables**

**Table S1. Simulated DRP1 complexes and solvated-system composition.**

| **Ligand** | **Formal charge** | **Ligand atoms** | **Total atoms** | **Waters** | **Box edge (Å)** | **NaCl (M)** |
| --- | --- | --- | --- | --- | --- | --- |
| **GTP** | −4 | 44 | 63,683 | 19,471 | 97.25 | 0.1512 |
| **Mdivi-1** | 0 | 32 | 63,539 | 19,427 | 97.20 | 0.1516 |
| **Olanzapine** | 0 | 42 | 63,927 | 19,553 | 97.38 | 0.1506 |
| **Olanzapine-H+** | +1 | 43 | 63,938 | 19,556 | 97.40 | 0.1506 |
| **Drpitor1a** | 0 | 27 | 63,276 | 19,341 | 97.09 | 0.1523 |
| **i127** | +1 | 54 | 64,057 | 19,592 | 97.47 | 0.1503 |

**Table S2. Structural metrics over the full 10.0-ns production trajectory.**

| **System** | **Cα RMSD (Å)** | **Pocket bb RMSD (Å)** | **Pose RMSD (Å)** | **Internal RMSD (Å)** | **COM drift (Å)** | **RMSF (Å)** | **Buried fraction** |
| --- | --- | --- | --- | --- | --- | --- | --- |
| **GTP (control)** | 1.95 ± 0.32 | 1.18 | 1.58 | 0.78 | 1.16 | 0.98 | 0.85 |
| **Mdivi-1** | 2.70 ± 0.45 | 1.15 | 4.00 | 0.40 | 0.88 | 1.01 | 0.61 |
| **Olanzapine (neutral)** | 2.19 ± 0.29 | 1.79 | 16.01 | 0.64 | 12.69 | 1.10 | 0.22 |
| **Olanzapine-H⁺** | 2.94 ± 0.57 | 1.65 | 7.94 | 0.67 | 4.16 | 1.08 | 0.47 |
| **Drpitor1a** | 2.27 ± 0.57 | 1.52 | 8.44 | 0.18 | 5.35 | 1.19 | 0.52 |
| **i127** | 2.03 ± 0.25 | 1.62 | 8.16 | 1.44 | 5.51 | 1.10 | 0.60 |

**Table S3. GROMOS clustering sensitivity and pocket occupancy.**

| **System** | **Heavy atoms** | **Clusters 1.5 Å** | **Top-1 1.5 Å** | **Clusters 2.0 Å** | **Top-1 2.0 Å** | **Clusters 2.5 Å** | **Top-1 2.5 Å** | **Pocket occupancy** |
| --- | --- | --- | --- | --- | --- | --- | --- | --- |
| **GTP (control)** | 32 | 1 | 100.0% | 1 | 100.0% | 1 | 100.0% | 100.0% |
| **Mdivi-1** | 22 | 12 | 88.0% | 6 | 98.3% | 1 | 100.0% | 100.0% |
| **Olanzapine (neutral)** | 22 | 451 | 4.6% | 283 | 7.8% | 175 | 11.2% | 6.9% |
| **Olanzapine-H⁺** | 22 | 111 | 28.5% | 51 | 39.8% | 32 | 47.1% | 57.1% |
| **Drpitor1a** | 19 | 100 | 31.1% | 46 | 35.6% | 29 | 43.5% | 53.4% |
| **i127** | 27 | 58 | 26.9% | 32 | 39.2% | 19 | 61.8% | 92.0% |

**Table S4. Key ligand-protein interaction occupancies (contacts with occupancy ≥0.15).**

| **System** | **Residue** | **Interaction** | **Occupancy** |
| --- | --- | --- | --- |
| GTP (control) | Asp218 | charged | 1.000 |
| GTP (control) | Lys38 | charged | 0.981 |
| GTP (control) | Ser40 | H-bond | 0.976 |
| GTP (control) | Lys216 | charged | 0.922 |
| GTP (control) | Asp218 | H-bond | 0.892 |
| GTP (control) | Arg247 | H-bond | 0.880 |
| GTP (control) | Arg53 | charged | 0.773 |
| GTP (control) | Ile252 | hydrophobic | 0.745 |
| GTP (control) | Ser36 | H-bond | 0.731 |
| GTP (control) | Lys38 | H-bond | 0.702 |
| GTP (control) | Asn246 | H-bond | 0.697 |
| GTP (control) | Leu219 | hydrophobic | 0.663 |
| GTP (control) | Gln34 | H-bond | 0.253 |
| GTP (control) | Arg53 | H-bond | 0.219 |
| Mdivi-1 | Ile252 | hydrophobic | 0.899 |
| Mdivi-1 | Arg53 | charged | 0.359 |
| Olanzapine (neutral) | Ile252 | hydrophobic | 0.175 |
| Olanzapine-H⁺ | Ile252 | hydrophobic | 0.914 |
| Olanzapine-H⁺ | Leu219 | hydrophobic | 0.178 |
| Drpitor1a | Ile252 | hydrophobic | 0.768 |
| Drpitor1a | Val58 | hydrophobic | 0.508 |
| Drpitor1a | Glu43 | charged | 0.322 |
| Drpitor1a | Arg53 | charged | 0.200 |
| Drpitor1a | Glu43 | H-bond | 0.174 |
| i127 | Asp218 | charged | 0.959 |
| i127 | Leu219 | hydrophobic | 0.934 |
| i127 | Ile252 | hydrophobic | 0.865 |
| i127 | Asp218 | H-bond | 0.753 |
| i127 | Ser40 | H-bond | 0.173 |

**Table S5. Catalytic Mg2+ coordination over the full 10.0 ns.**

| **System** | **Mg–Ser39 OG (Å)** | **Mg–ligand min (Å)** | **Inner-shell waters** | **Ser39  inner-shell occupancy** | **Ligand  inner-shell occupancy** |
| --- | --- | --- | --- | --- | --- |
| **GTP (control)** | 2.19 | 1.88 | 2.00 | 0.999 | 1.00 |
| **Mdivi-1** | 6.12 | 13.36 | 4.00 | 0.000 | 0.00 |
| **Olanzapine (neutral)** | 4.39 | 18.93 | 4.04 | 0.000 | 0.00 |
| **Olanzapine-H⁺** | 5.17 | 13.53 | 4.72 | 0.000 | 0.00 |
| **Drpitor1a** | 4.41 | 9.77 | 4.00 | 0.000 | 0.00 |
| **i127** | 5.33 | 13.40 | 4.95 | 0.000 | 0.00 |

**Table S6. MM/PBSA components at εin = 2. Values are kcal mol-1; ΔGbind contains no entropy term and SE is autocorrelation-corrected.**

| **System** | **N** | **ΔEvdW** | **ΔEelec** | **ΔGPB** | **ΔGnonpolar** | **ΔGbind** | **SE** | **95% CI** |
| --- | --- | --- | --- | --- | --- | --- | --- | --- |
| **GTP (control)** | 200 | −20.97 | −792.22 | 689.89 | −4.11 | −127.41 | ± 4.86 | −141.62 to −113.21 |
| **Mdivi-1** | 200 | −26.63 | −5.28 | 15.86 | −3.09 | −19.14 | ± 0.46 | −20.07 to −18.21 |
| **Olanzapine (neutral)** | 200 | −10.35 | −1.56 | 4.23 | −1.38 | −9.06 | ± 1.61 | −12.69 to −5.43 |
| **Olanzapine-H⁺** | 200 | −22.91 | 2.36 | 4.06 | −2.69 | −19.18 | ± 1.56 | −22.92 to −15.44 |
| **Drpitor1a** | 200 | −20.25 | −4.11 | 10.75 | −2.25 | −15.86 | ± 1.05 | −18.35 to −13.38 |
| **i127** | 200 | −31.16 | 3.90 | 6.25 | −3.33 | −24.33 | ± 1.35 | −27.09 to −21.57 |

**Table S7. MM/PBSA components at εin = 4. Values are kcal mol-1; ΔGbind contains no entropy term and SE is autocorrelation-corrected.**

| **System** | **N** | **ΔEvdW** | **ΔEelec** | **ΔGPB** | **ΔGnonpolar** | **ΔGbind** | **SE** | **95% CI** |
| --- | --- | --- | --- | --- | --- | --- | --- | --- |
| **GTP (control)** | 200 | −20.97 | −396.11 | 330.47 | −4.11 | −90.73 | ± 3.18 | −99.21 to −82.24 |
| **Mdivi-1** | 200 | −26.63 | −2.64 | 7.86 | −3.09 | −24.50 | ± 0.45 | −25.43 to −23.57 |
| **Olanzapine (neutral)** | 200 | −10.35 | −0.78 | 2.09 | −1.38 | −10.41 | ± 1.90 | −14.72 to −6.10 |
| **Olanzapine-H⁺** | 200 | −22.91 | 1.18 | 2.16 | −2.69 | −22.26 | ± 1.75 | −26.44 to −18.09 |
| **Drpitor1a** | 200 | −20.25 | −2.06 | 5.37 | −2.25 | −19.18 | ± 1.46 | −23.04 to −15.33 |
| **i127** | 200 | −31.16 | 1.95 | 3.62 | −3.33 | −28.91 | ± 1.41 | −31.79 to −26.04 |

**Table S8. Multiplicity-corrected pairwise ΔΔG contrasts among the five commensurable small molecules.**

| **Contrast** | **ΔΔG (ε_in_ = 2)** | **q (ε_in_ = 2)** | **ΔΔG (ε_in_ = 4)** | **q (ε_in_ = 4)** |
| --- | --- | --- | --- | --- |
| **olanzapine → olanzapine-H+** | 10.12 | < 0.001 | 11.85 | < 0.001 |
| **Mdivi-1 → olanzapine-H+** | 0.04 | 0.982 | −2.24 | 0.252 |
| **Mdivi-1 → olanzapine** | −10.08 | < 0.001 | −14.09 | < 0.001 |
| **Mdivi-1 → Drpitor1a** | −3.27 | 0.024 | −5.32 | 0.021 |
| **Mdivi-1 → i127** | 5.19 | 0.002 | 4.41 | 0.009 |
| **olanzapine-H+ → Drpitor1a** | −3.31 | 0.122 | −3.08 | 0.237 |
| **i127 → Drpitor1a** | −8.47 | < 0.001 | −9.73 | < 0.001 |

**Table S9. Autocorrelation-corrected sampling statistics for ΔGbind.**

| **System** | **N** | **τ_int_ (ε_in_ = 2)** | **Neff (ε_in_ = 2)** | **SE corr. (ε_in_ = 2)** | **SE naive (ε_in_ = 2)** | **SE block (ε_in_ = 2)** | **τ_int_ (ε_in_ = 4)** | **Neff (ε_in_ = 4)** |
| --- | --- | --- | --- | --- | --- | --- | --- | --- |
| **GTP (control)** | 200 | 22.03 | 4.5 | 4.86 | 0.73 | 2.59 | 18.31 | 5.5 |
| **Mdivi-1** | 200 | 2.95 | 33.9 | 0.46 | 0.19 | 0.32 | 3.54 | 28.2 |
| **Olanzapine (neutral)** | 200 | 9.63 | 10.4 | 1.61 | 0.37 | 1.21 | 10.21 | 9.8 |
| **Olanzapine-H⁺** | 200 | 13.38 | 7.5 | 1.56 | 0.30 | 0.99 | 13.11 | 7.6 |
| **Drpitor1a** | 200 | 12.32 | 8.1 | 1.05 | 0.21 | 0.53 | 17.73 | 5.6 |
| **i127** | 200 | 3.44 | 29.1 | 1.35 | 0.51 | 1.03 | 3.20 | 31.2 |

**Table S10. Strongest attractive receptor-residue decomposition terms at εin = 4. The threshold is a reporting convention, not a significance test; for the cationic ligands, long-range electrostatics can dominate residues outside the binding pocket.**

| **System** | **Residue** | **Mean total (kcal mol-1)** | **SD across 5 shard means** |
| --- | --- | --- | --- |
| GTP (control) | MG323 | -370.70 | 3.83 |
| GTP (control) | LYS38 | -123.88 | 8.85 |
| GTP (control) | ARG53 | -73.44 | 14.98 |
| GTP (control) | LYS216 | -71.20 | 3.07 |
| GTP (control) | LYS152 | -41.83 | 6.43 |
| GTP (control) | ARG247 | -41.19 | 2.19 |
| GTP (control) | ARG108 | -35.52 | 3.54 |
| GTP (control) | ARG167 | -33.85 | 1.21 |
| Mdivi-1 | ARG53 | -4.02 | 2.10 |
| Mdivi-1 | GLN249 | -3.21 | 0.62 |
| Mdivi-1 | ASP218 | -2.28 | 0.54 |
| Mdivi-1 | ASN246 | -1.59 | 0.45 |
| Mdivi-1 | SER248 | -1.28 | 0.40 |
| Drpitor1a | ARG53 | -4.02 | 1.02 |
| Drpitor1a | GLU43 | -2.05 | 2.19 |
| Drpitor1a | ASP49 | -1.36 | 0.93 |
| i127 | ASP218 | -20.42 | 4.31 |
| i127 | GLU43 | -13.90 | 3.04 |
| i127 | ASP49 | -11.60 | 2.45 |
| i127 | ASP251 | -11.59 | 2.28 |
| i127 | GLU265 | -8.77 | 1.68 |
| i127 | ASP146 | -8.45 | 1.65 |
| i127 | ASP264 | -7.60 | 1.42 |
| i127 | ASP105 | -7.35 | 1.34 |
| olanzapine | ARG53 | -2.33 | 2.59 |
| olanzapine-H+ | GLU43 | -10.75 | 1.18 |
| olanzapine-H+ | ASP49 | -10.10 | 1.41 |
| olanzapine-H+ | GLU112 | -9.60 | 1.98 |
| olanzapine-H+ | ASP251 | -8.50 | 0.62 |
| olanzapine-H+ | ASP105 | -8.49 | 1.44 |
| olanzapine-H+ | ASP218 | -8.14 | 1.42 |
| olanzapine-H+ | ASP146 | -7.67 | 1.01 |
| olanzapine-H+ | GLU116 | -6.84 | 0.81 |

**Supplementary Results**

**S7. Assembly and equilibration quality control**

All six receptor coordinate sets were identical at the heavy-atom level, and all assembled complexes were free of severe ligand-receptor clashes. Solvated systems were neutral to numerical precision and converged to similar NPT densities near 1.01 g cm-3. The early production ensemble audit covered only 1.5-2.0 ns of each 10-ns trajectory and was not regenerated for the final 8 ns; by contrast, solute continuity and finite-valued structural analyses were verified over all 1,000 frames. This distinction is important: the trajectory dataset is complete, but the formal thermostat/barostat audit is partial.

**S8. Ligand retention and conformational heterogeneity**

GTP remained in a single crystallographic-like cluster and Mdivi-1 maintained 100% pocket occupancy with a highly populated dominant binding mode. Protonated OLZ, Drpitor1a, and DRP1i27 shifted from their docked starting poses but remained associated with the receptor to varying degrees. Neutral OLZ was qualitatively different: pocket occupancy fell to 6.9%, mean COM drift reached 12.69 Å, and its final buried fraction was zero. Cluster-count sensitivity did not alter this binary conclusion: neutral OLZ leaves, whereas the other small molecules remain associated at the end of production.

**S9. Interaction fingerprints and catalytic Mg2+**

The GTP control retained the expected catalytic network involving the P-loop and guanine-recognition motifs. Mdivi-1 and protonated OLZ instead relied on hydrophobic Ile252 (0.899 and 0.914 occupancy) without a persistent hydrogen-bond network. DRP1i27 partially reproduced a GTP-like Asp218 interaction, whereas the other inhibitors did not. Only GTP retained inner-shell coordination to Mg2+ and Ser39; all five inhibitor systems showed 0% inner-shell occupancy and a more highly hydrated Mg2+ ion.

**S10. MM/PBSA ranking, dielectric sensitivity, and statistical contrasts**

The within-inhibitor ranking was largely preserved between ε_in_ = 2 and 4 (Spearman ρ = 0.94), with only Mdivi-1 and protonated OLZ exchanging second and third positions. That exchange occurred precisely for the pair that could not be distinguished statistically at either dielectric. The protonation contrast for OLZ was robust and large at both dielectric values, whereas Mdivi-1 versus protonated OLZ remained unresolved. Effective sample sizes were only 4.5-33.9 despite 200 snapshots because the MM/PBSA time series were autocorrelated; corrected standard errors were several-fold larger than naive snapshot-based errors. The GTP control was retained as a qualitative positive control rather than an affinity comparator because the formal -4 ligand and retained divalent cation produce very large and strongly dielectric-sensitive continuum electrostatics.

**S11. Per-residue decomposition and stability-affinity relationship**

Per-residue decomposition at ε_in_ = 4 is useful as a cross-check but not as a map of physical contacts for the charged systems. For protonated OLZ and DRP1i27, the most attractive terms include distant acidic residues that are not geometrically contacted, reflecting long-range Coulomb contributions. Consequently, the main manuscript bases residue-level mechanistic claims on geometric occupancies. Across the five inhibitors, correlations between MM/PBSA rank and pose RMSD (ρ = 0.70, p = 0.188), COM drift (ρ = 0.40, p = 0.505), or pocket-backbone RMSD (ρ = 0.50, p = 0.391) were descriptive and non-significant at n=5; the complete trajectories therefore do not establish that more stable poses necessarily have more favorable MM/PBSA values.

**Supplementary Figures**

**Supplementary Figure S1. Unified DRP1 complex assembly quality control. The six systems use the same 4H1V-derived receptor and catalytic Mg2+. Panels summarize steric validation, nucleotide-pocket contacts in the starting structures, the initial Mg2+ environment, and receptor-coordinate identity.**

**Supplementary Figure S2. NVT equilibration at 310.15 K. Temperature, density/box volume (where applicable), and potential energy are shown for the six systems during the restrained constant-volume heating/equilibration stage.**

**Supplementary Figure S3. NPT equilibration at 310.15 K and 1 bar. Temperature, density, potential energy, and box-volume traces show relaxation of the solvated systems before unrestrained pre-equilibration.**

**Supplementary Figure S4. Relaxation of the catalytic Mg2+ environment after equilibration. Only the native GTP control retains the crystallographic Mg2+-Ser39 inner-sphere contact; the inhibitor systems become solvent-coordinated.**

**Supplementary Figure S5. Conformational stability over the complete 10.0-ns production trajectories. The panels show global and pocket RMSD, ligand pose and internal RMSD, center-of-mass drift, protein radius of gyration, ligand SASA/buried fraction, and second-half global RMSD distributions.**

**Supplementary Figure S6. Per-residue ΔRMSF relative to the GTP/Mg2+ control over the full production trajectory. Colored bars indicate greater flexibility and gray bars rigidification; canonical G-domain motifs are shaded.**

**Supplementary Figure S7. Ligand-protein interaction occupancy over all 1,000 production frames, separated into hydrogen-bond, hydrophobic, and charged-contact maps. The Ile252 hydrophobic column is the principal shared anchor across the small-molecule panel.**

**Supplementary Figure S8. Catalytic Mg2+ coordination over the full production trajectories. GTP alone remains within the 2.6-Å inner-shell criterion; the inhibitor complexes retain a solvent-separated Mg2+ environment.**

**Supplementary Figure S9. Sensitivity of the MM/PBSA estimator to the solute dielectric. Absolute values shift from εin=2 to 4, while the inhibitor ranking is largely stable; the only rank exchange is Mdivi-1 versus protonated OLZ.**

**Supplementary Figure S10. Per-residue MM/PBSA decomposition at εin=4. The strongest attractive receptor terms are ranked within each system; for the cationic ligands the leading terms are dominated by long-range Coulomb attraction and should not be equated with physical pocket contacts.**

**Supplementary Figure S11. Shared pocket anchors: geometric occupancy and per-residue decomposed energies. Ile252 is the near-identical high-occupancy anchor for Mdivi-1 and protonated OLZ, whereas decomposition illustrates why charged-ligand residue energies can be misleading in isolation.**

**Supplementary Figure S12. Pose stability versus MM/PBSA interaction energy for the five inhibitor complexes. Correlations are descriptive only (n=5) and do not reach statistical significance over the completed 10-ns dataset.**

**Computational limitations and deviations**

Production used 4-fs hydrogen-mass-repartitioned dynamics rather than the planned 2-fs timestep, without a 2-fs control. The formal production thermodynamic-ensemble QC was generated at an early checkpoint and covers only 1.5-2.0 ns, although structural integrity was checked over the complete trajectories. Drpitor1a and DRP1i27 did not satisfy the separate pre-equilibration settling criterion. No replicate trajectories were run; no direct dual-ligand competition or steric-overlap analysis was performed; MM/PBSA omitted entropy and used a single-trajectory approximation; and per-residue decomposition was evaluated only at εin=4. These limitations restrict the simulations to hypothesis refinement and relative comparison rather than experimental-affinity determination.

**Supplementary References**

Chodera, J. D. (2016). A simple method for automated equilibration detection in molecular simulations. Journal of Chemical Theory and Computation 12, 1799-1805. doi: 10.1021/acs.jctc.5b00784

Darden, T., York, D., and Pedersen, L. (1993). Particle mesh Ewald: an N·log(N) method for Ewald sums in large systems. Journal of Chemical Physics 98, 10089-10092. doi: 10.1063/1.464397

Daura, X., Gademann, K., Jaun, B., Seebach, D., van Gunsteren, W. F., and Mark, A. E. (1999). Peptide folding: when simulation meets experiment. Angewandte Chemie International Edition 38, 236-240. doi: 10.1002/(SICI)1521-3773(19990115)38:1/2<236::AID-ANIE236>3.0.CO;2-M

Eastman, P., Galvelis, R., Peláez, R. P., et al. (2024). OpenMM 8: molecular dynamics simulation with machine learning potentials. Journal of Physical Chemistry B 128, 109-116. doi: 10.1021/acs.jpcb.3c06662

Eastman, P., Swails, J., Chodera, J. D., et al. (2017). OpenMM 7: rapid development of high performance algorithms for molecular dynamics. PLOS Computational Biology 13, e1005659. doi: 10.1371/journal.pcbi.1005659

Flyvbjerg, H., and Petersen, H. G. (1989). Error estimates on averages of correlated data. Journal of Chemical Physics 91, 461-466. doi: 10.1063/1.457480

Genheden, S., and Ryde, U. (2015). The MM/PBSA and MM/GBSA methods to estimate ligand-binding affinities. Expert Opinion on Drug Discovery 10, 449-461. doi: 10.1517/17460441.2015.1032936

Grossfield, A., Patrone, P. N., Roe, D. R., Schultz, A. J., Siderius, D., and Zuckerman, D. M. (2019). Best practices for quantification of uncertainty and sampling quality in molecular simulations. Living Journal of Computational Molecular Science 1, 5067. doi: 10.33011/livecoms.1.1.5067

Hopkins, C. W., Le Grand, S., Walker, R. C., and Roitberg, A. E. (2015). Long-time-step molecular dynamics through hydrogen mass repartitioning. Journal of Chemical Theory and Computation 11, 1864-1874. doi: 10.1021/ct5010406

Jakalian, A., Jack, D. B., and Bayly, C. I. (2002). Fast, efficient generation of high-quality atomic charges. AM1-BCC model: II. Parameterization and validation. Journal of Computational Chemistry 23, 1623-1641. doi: 10.1002/jcc.10128

Jorgensen, W. L., Chandrasekhar, J., Madura, J. D., Impey, R. W., and Klein, M. L. (1983). Comparison of simple potential functions for simulating liquid water. Journal of Chemical Physics 79, 926-935. doi: 10.1063/1.445869

Joung, I. S., and Cheatham, T. E. (2008). Determination of alkali and halide monovalent ion parameters for use in explicitly solvated biomolecular simulations. Journal of Physical Chemistry B 112, 9020-9041. doi: 10.1021/jp8001614

Li, P., Roberts, B. P., Chakravorty, D. K., and Merz, K. M. (2013). Rational design of particle mesh Ewald compatible Lennard-Jones parameters for +2 metal cations in explicit solvent. Journal of Chemical Theory and Computation 9, 2733-2748. doi: 10.1021/ct400146w

Maier, J. A., Martinez, C., Kasavajhala, K., Wickstrom, L., Hauser, K. E., and Simmerling, C. (2015). ff14SB: improving the accuracy of protein side chain and backbone parameters from ff99SB. Journal of Chemical Theory and Computation 11, 3696-3713. doi: 10.1021/acs.jctc.5b00255

Meagher, K. L., Redman, L. T., and Carlson, H. A. (2003). Development of polyphosphate parameters for use with the AMBER force field. Journal of Computational Chemistry 24, 1016-1025. doi: 10.1002/jcc.10262

Rosdah, A. A., Abbott, B. M., Langendorf, C. G., Deng, Y., Truong, J. Q., Waddell, H. M. M., et al. (2022). A novel small molecule inhibitor of human Drp1. Sci. Rep. 12:21531. doi: 10.1038/s41598-022-25464-z

Sun, H., Li, Y., Shen, M., et al. (2014). Improved docking performance using high solute dielectric constant MM/GBSA and MM/PBSA rescoring. Physical Chemistry Chemical Physics 16, 22035-22045. doi: 10.1039/C4CP03179B

Wang, E., Sun, H., Wang, J., et al. (2019). End-point binding free energy calculation with MM/PBSA and MM/GBSA: strategies and applications in drug design. Chemical Reviews 119, 9478-9508. doi: 10.1021/acs.chemrev.9b00055

Wang, J., Wolf, R. M., Caldwell, J. W., Kollman, P. A., and Case, D. A. (2004). Development and testing of a general AMBER force field. Journal of Computational Chemistry 25, 1157-1174. doi: 10.1002/jcc.20035

Wenger, J., Klinglmayr, E., Fröhlich, C., Eibl, C., Gimeno, A., Hessenberger, M., et al. (2013). Functional mapping of human dynamin-1-like GTPase domain based on X-ray structure analyses. PLoS ONE 8:e71835. doi: 10.1371/journal.pone.0071835

Wu, D., Dasgupta, A., Chen, K. H., Neuber-Hess, M., Patel, J., Hurst, T. E., et al. (2020). Identification of novel dynamin-related protein 1 (Drp1) GTPase inhibitors: Therapeutic potential of Drpitor1 and Drpitor1a in cancer and cardiac ischemia-reperfusion injury. FASEB J. 34, 1447-1464. doi: 10.1096/fj.201901467R
