# Supplementary material for: Olanzapine induces Mdivi-1-resistant disruption of insulin signaling in skeletal muscle cells
Source: Front Pharmacol. 2026 Oct 26;17:1920978. doi: 10.3389/fphar.2026.1920978 (PMC13745686; doi:10.3389/fphar.2026.1920978)
Supplement: Supplementary file 2 [file Table1.docx]

**Supplementary figure 1**

**Figure 1 - panel D**

| Control vs.  OLZ | 0.0363* |
| --- | --- |
| Control vs. Mdivi-1 1 µM | >0.9999 |
| Control vs. Mdivi-1 1 µM + OLZ | 0.1266 |
| Control vs. Mdivi-1 10 µM | >0.9999 |
| Control vs. Mdivi-1 10 µM + OLZ | 0.1737 |
| OLZ vs. Mdivi-1 1 µM | 0.0125* |
| OLZ vs. Mdivi-1 1 µM + OLZ | >0.9999 |
| OLZ vs. Mdivi-1 10 µM | 0.0460* |
| OLZ vs. Mdivi-1 10 µM + OLZ | >0.9999 |
| Mdivi-1 1 µM vs. Mdivi-1 1 µM + OLZ | 0.0420* |
| Mdivi-1 1 µM vs. Mdivi-1 10 µM | >0.9999 |
| Mdivi-1 1 µM vs. Mdivi-1 10 µM + OLZ | 0.0574 |
| Mdivi-1 1 µM + OLZ vs. Mdivi-1 10 µM | 0.1611 |
| Mdivi-1 1 µM + OLZ vs. Mdivi-1 10 µM + OLZ | >0.9999 |
| Mdivi-1 10 µM vs. Mdivi-1 10 µM + OLZ | 0.2212 |

**Figure 2 - panel B**

| Control vs. Insulin | 0.0009*** |
| --- | --- |
| OLZ vs. OLZ + Insulin | 0.1948 |
| Mdivi-1 vs. Mdivi-1 + Insulin | 0.1940 |
| OLZ + Mdivi-1 vs. OLZ + Mdivi-1 + Insulin | >0.9999 |

**Figure 2 - panel C**

| Control vs. Insulin | 0.0309* |
| --- | --- |
| OLZ vs. OLZ + Insulin | 0.1931 |
| Mdivi-1 vs. Mdivi-1 + Insulin | 0.0209* |
| OLZ + Mdivi-1 vs. OLZ + Mdivi-1 + Insulin | >0.9999 |

**Figure 3 - panel C**

| Control vs. Insulin | 0.0156* |
| --- | --- |
| OLZ vs. OLZ + Insulin | 0.2106 |
| Mdivi-1 vs. Mdivi-1 + Insulin | 0.0009*** |
| OLZ + Mdivi-1 vs. OLZ + Mdivi-1 + Insulin | 0.1244 |

**Figure 4 - panel A**

| Control vs. Insulin | 0.2026 |
| --- | --- |
| OLZ vs. OLZ + Insulin | 0.6204 |
| Mdivi-1 vs. Mdivi-1 + Insulin | >0.9999 |
| OLZ + Mdivi-1 vs. OLZ + Mdivi-1 + Insulin | >0.9999 |

**Figure 4 - panel D**

| Control vs. OLZ | 0.0133* |
| --- | --- |
| Control vs. Mdivi-1 | 0.0965 |
| Control vs. OLZ + Mdivi-1 | <0.0001**** |
| OLZ vs. Mdivi-1 | <0.0001**** |
| OLZ vs. OLZ + Mdivi-1 | 0.0031** |
| Mdivi-1 vs. OLZ + Mdivi-1 | <0.0001**** |
| Insulin vs. OLZ + Insulin | 0.0072** |
| Insulin vs. Mdivi-1 + Insulin | 0.5085 |
| Insulin vs. OLZ + Mdivi-1 + Insulin | <0.0001**** |
| OLZ + Insulin vs. Mdivi-1 + Insulin | 0.0002*** |
| OLZ + Insulin vs. OLZ + Mdivi-1 + Insulin | 0.0043** |
| Mdivi-1 + Insulin vs. OLZ + Mdivi-1 + Insulin | <0.0001**** |
| Control vs. CCCP | 0.0413* |

**Figure 4 - panel E**

| Control vs. Insulin | 0.1987 |
| --- | --- |
| OLZ vs. OLZ + Insulin | >0.9999 |
| Mdivi-1 vs. Mdivi-1 + Insulin | 0.2226 |
| OLZ + Mdivi-1 vs. OLZ + Mdivi-1 + Insulin | >0.9999 |

**Figure 5 - panel B**

| Control vs. OLZ | <0.0001**** |
| --- | --- |
| Control vs. Mdivi-1 | 0.2610 |
| Control vs. Mdivi-1 + OLZ | 0.0018** |

**Figure 5 - panel C**

| Control vs. OLZ | >0.9999 |
| --- | --- |
| Control vs. Mdivi-1 | 0.2787 |
| Control vs. Mdivi-1 + OLZ | 0.0353* |

**Figure 5 - panel D**

| Control vs. OLZ | 0.0001*** |
| --- | --- |
| Control vs. Mdivi-1 | >0.9999 |
| Control vs. Mdivi-1 + OLZ | 0.1116 |

**Figure 5 - panel E**

| Control vs. OLZ | >0.9999 |
| --- | --- |
| Control vs. Mdivi-1 | >0.9999 |
| Control vs. Mdivi-1 + OLZ | 0.3023 |
